# Regulating the microporous structure by cavity-shaped molecular sieving channels

**DOI:** 10.1126/sciadv.aee9782

**Published:** 2026-07-29

**Authors:** Wen He, Xuan Ding, Ali A. AL-Thuraya, Daijun Meng, Bizi Yu, Wuyueming Zhou, Yumo Fan, Yuting Zhang, Hongjun Zhang, Jiangtao Liu

**Affiliations:** ^1^ State Key Laboratory of Advanced Environmental Technology, School of Environment, University of Science and Technology of China, Hefei 230026, China.; ^2^ State Key Laboratory of Particle Detection and Electronics, University of Science and Technology of China, Hefei 230026, China.

## Abstract

Membrane separation technology represents a promising and effective alternative to traditional energy-intensive gas separation technologies. However, most commercial membranes have low permeability and moderate selectivity, which limits their ability to meet the stringent gas purity requirements for hydrogen purification and natural gas upgrading. In this study, Matrimid, a widely used commercial polymer, was selected as the matrix material. Cucurbituril (CB) with selective cavity was incorporated into Matrimid as short-path gas transport channels. This modification increased the gas transport pathways and enhanced the connectivity of gas transmission networks within the membrane structure. The H_2_ permeability of Matrimid-CB-5% membrane decreased from 113 to 74 Barrer, while the H_2_/CH_4_ selectivity increased from 169 to 743 during 600-day aging. Notably, the membranes achieved separation efficiencies that surpassed the latest upper bound line for CO_2_/N_2_ and CO_2_/CH_4_ separations. These results indicate a substantial improvement over widely used commercial materials such as Matrimid, cellulose acetate, polysulfone, and other mixed matrix membranes.

## Teaser

CB-Matrimid hydrogen bonding boosts compatibility, while CB cavities provide fast channels countering aging-induced decline.

## INTRODUCTION

Chemical separation, including distillation, evaporation, and drying in industrial processes and other thermally driven industrial separation processes, accounts for 10 to 15% of global energy consumption (*1*, *2*). Membrane separation technology can be an order of magnitude more energy efficient than highly energy-intensive heat-driven separation technologies and could potentially allow up to 90% energy savings (*3*). Membranes are favored over other processes for the purification of many critical gases due to their advantages in energy efficiency, simplicity, manufacturing scalability, and small footprint (*4*, *5*). Easily processable polymer membranes with high permeability and selectivity have become a critical material to achieve the high productivity and purity of gas products in the practical industrial applications (*6*), including hydrogen recovery (*7*–*9*), carbon capture from flue gas (*10*–*12*), and natural gas purification (*13*, *14*). However, it is a great challenge to further improve the gas transfer efficiency since traditional polymeric membranes are subject to an inherent trade-off relationship between gas permeability and selectivity (*15*–*17*).

Now, gas separation membranes are mostly relied on polymeric materials with customizable gas transport properties. The certain structural changes in these polymers regulate the free-volume distribution, which contribute to higher permeability-selectivity combinations (*18*–*20*). The dominant methods for modifying and controlling the structure of polymers are mainly cross-linking (*21*, *22*), high temperature treatment (*23*, *24*), and addition of nanofillers (*25*–*27*). The cross-linking method improves the gas selectivity by splitting the original large pore size and shrinking the polymer chain spacing, but it also faces issues such as harsh chemicals, high energy costs, and process complexity (*28*). The high temperature treatment method achieves shrinkage of the original pore size by pyrolytic rearrangement of polymer chains, and thus, thermally rearranged membranes and carbon molecular sieve membranes have very attractive gas transport properties, but the pyrolysis procedure consumes substantial energy and requires precise control (*29*). Mixed matrix membranes (MMMs) prepared by incorporating nanofillers with specific pore sizes into a processable polymer matrix have good processability, high stability, and excellent gas separation capacity (*30*). In addition, the method of adding fillers to polymers is easier to achieve mass production than other methods due to its easy preparation process, low cost, and good solution processability (*31*–*33*).

The design of gas separation membranes with ideal molecular sieving capability remains a daunting task owing to the very small sub-angstrom–level differences between gas molecules. For example, in the flue gas carbon capture, the kinetic diameters of CO_2_ and N_2_ are 3.30 and 3.64 Å, respectively, the size difference between the two is very small (<0.4 Å), and in the range of fine-tuning pore size (0.3 to 0.4 Å), any defects within the membrane could cause a catastrophic effect, seriously reducing its ability to separate the gas (*34*). Fields like hydrogen recovery and natural gas purification have high requirements on the separation selectivity of membranes to ensure the high gas purity (*35*). High permeability polymers are difficult to provide the necessary selectivity for high purity gas recovery. Commercial gas separation membranes usually have moderate selectivity but low permeability (*36*). Materials, such as polysulfone, polyimide, and cellulose acetate, are commonly used as polymer matrices (*37*–*39*). Therefore, in this work, Matrimid was selected among a variety of polymer materials, which is a type of polymer with dense chain stacking, and it can be used for the separation and purification of a variety of gas pairs. In addition, it is capable of maintaining stability of separation performance in harsh environments such as high temperature and high pressure (*40*, *41*).

Supramolecular macrocyclic compounds with only two windows have been used in various applications from CO_2_ capture to gas purification (*42*, *43*). Supramolecular macrocyclic compounds, such as crown ethers (*44*), cyclodextrins (*45*), cyclen (*46*), and calixarenes (*47*), have been incorporated into polymer matrices as organic fillers, and the polymer chain stacking is adjusted by intermolecular forces to achieve precise regulation of pore structure. Here, cucurbituril (CB), a supramolecular macrocycle with two equally sized windows, was chosen. It has a rigid, highly symmetric structure consisting of six repeating glycuronium units connected by 12 methylene bridges (*48*, *49*). The pumpkin-shaped CB macrocycle features an open intramolecular cavity with two hydrophilic, negatively charged cavity openings formed by carbonyl groups at the cavity periphery, while the central inner cavity is hydrophobic (*50*, *51*). Because of its unique property, CB is commonly used for the separation of contaminants from water. The structural features of CB are demonstrated in Fig. 1A. Its port diameter corresponds to 3.9 Å, and it has a cavity with a maximum diameter of 5.8 Å and a height of 9.1 Å (*48*). This cavity with a certain size is well suited as a short-range transport channel for the separation of critical gases to increase gas transfer efficiency (Fig. 1B).

**Fig. 1.**
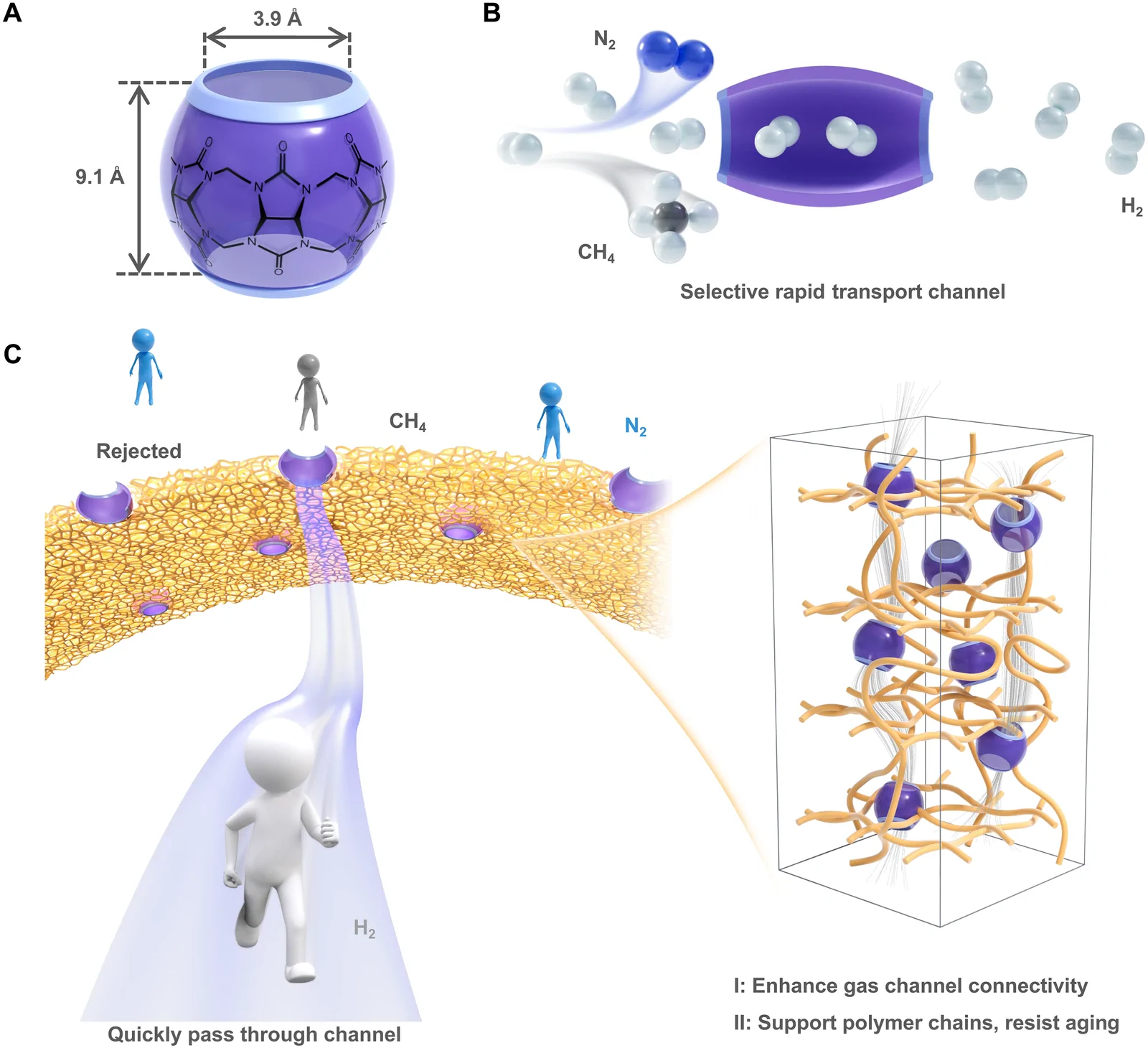
Schematic of the Matrimid-CB membrane for hydrogen purification. (**A**) Chemical structure of CB. (**B**) Cross section of CB. (**C**) Hydrogen rapid transport channels via CB in Matrimid-CB membrane.

In this work, we incorporated CB, which has a gas-selective window and short-range transport channel, into Matrimid to enhance the gas transfer efficiency and separation performance. Here, CB can form hydrogen bonding with Matrimid chains (fig. S1), enhance the interfacial compatibility of the Matrimid-CB membranes, and reduce the number of nonselective pores within the membrane. The cavity of CB with a certain size acts as a short-range transport channel for small gas molecules, enhancing the connectivity of gas transport pathways, thereby substantially improving the gas transport efficiency and screening capabilities of Matrimid-CB membranes (Fig. 1C). In addition, the stable selective gas transport cavity structure of CB effectively resists the negative effects of increased chain stacking density during long-term aging, maintaining the permeability of small gas molecule transport pathways. As a result, the Matrimid-CB membrane exhibits superior anti-aging performance compared to other Matrimid-based MMMs and commercial gas separation membranes.

## RESULTS

### Fabrication and characterizations of Matrimid-CB membranes

Matrimid-CB membranes can be easily prepared by a solution casting method. Optical photography presents that Matrimid-CB membranes were yellow, uniform, and highly transparent, exhibiting a mirror-like smoothness (Fig. 2A). The microscopic morphology of the membranes has a notable impact on membrane performance. Scanning electron microscopy (SEM) cross sections of the membranes (Fig. 2B and fig. S2) show that the Matrimid membrane demonstrated dense and smooth morphology. For the Matrimid-CB membrane, the arcuate vein structures are randomly and uniformly distributed in the polymer matrix in a nonaligned manner. This uniform distribution of arcuate vein structures at high loading was realized by the subtle intermolecular forces between CB and Matrimid. Specifically, the favorable interactions between CB and Matrimid cause interfacial stresses during the fracturing of the MMMs in liquid nitrogen, which stretches and deforms the polymer segments to form an arcuate vein structure. The random and uniform distribution of the arcuate vein structure indicates that CB is also randomly and uniformly distributed in the membrane in a nonaligned manner. In the process of membrane formation by solvent evaporation, the subtle molecular interactions between CB and Matrimid enhanced the interfacial compatibility between two phases and eliminated some interfacial defects, which had a positive effect on the MMM performance. The high magnification SEM images (Fig. 2C and fig. S3) clearly show that both the membrane cross section and surface section exhibit a continuous phase, indicating that CB is well dispersed in the membrane without agglomeration, and the corresponding N-elements energy dispersive X-ray spectroscopy (EDS) elemental maps (Fig. 2D) further confirm the uniform distribution of CB in the polymer matrix. This allows CB to reduce the mass transfer resistance over short distances, providing a “percolation highway” for the selected gas molecules (i.e., H_2_ and CO_2_).

**Fig. 2.**
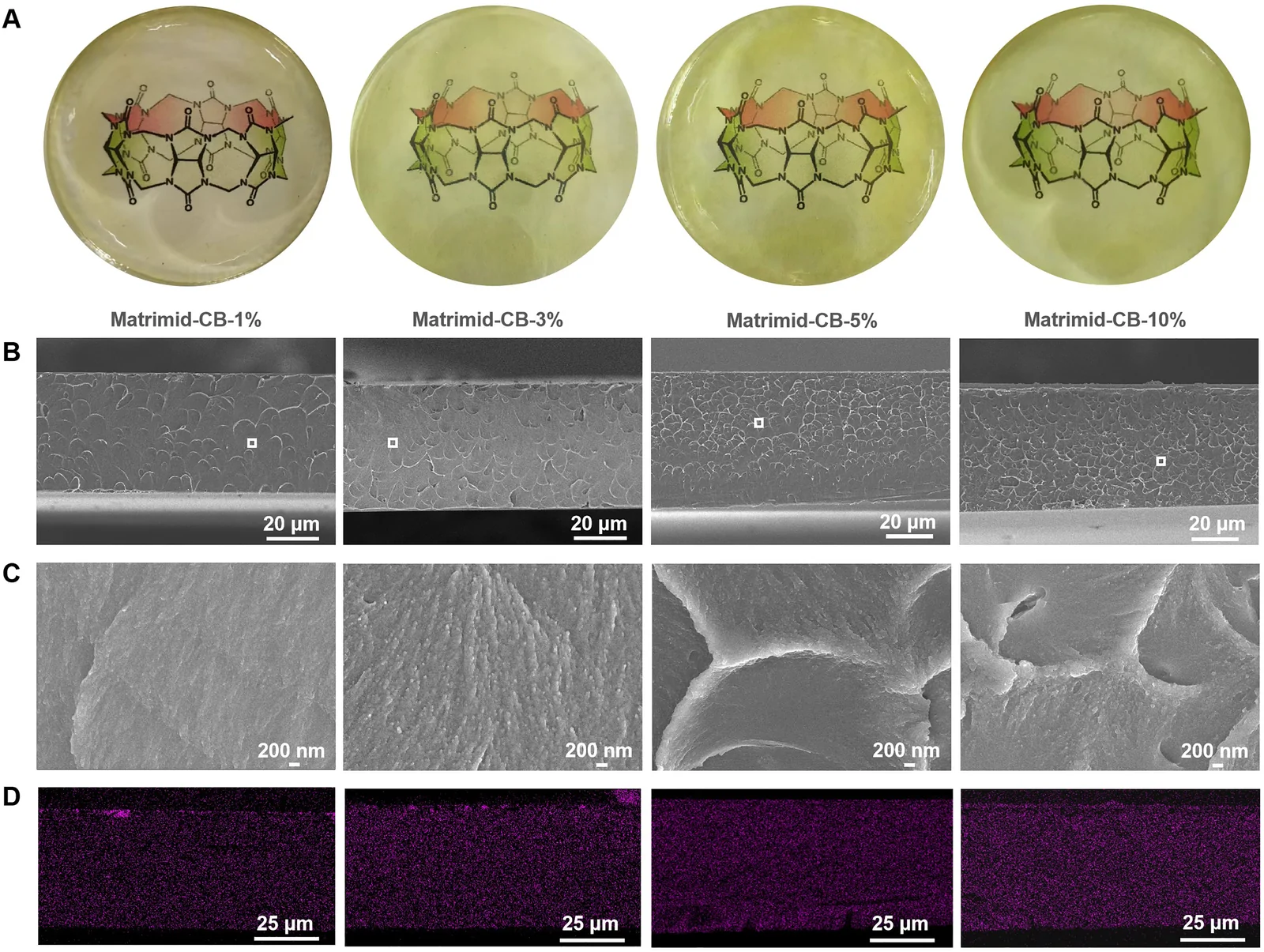
SEM images of the Matrimid-CB membranes. (**A**) Photograph and (**B** and **C**) cross section of the Matrimid-CB-*n*% membranes (*n*% is the CB mass loading and was varied from 1 to 10 wt %, *n* = 1, 3, 5, and 10). (**D**) Cross-sectional SEM-EDS nitrogen (N) mapping of Matrimid-CB membranes.

Figure 3A compares the x-ray diffraction (XRD) patterns of the Matrimid and Matrimid-CB membranes. Both of them exhibit two amorphous characteristic peaks of Matrimid nearby 15° and 22°. With increasing of CB content (1 to 3wt %) in the MMMs, the characteristic peak near 15° shifted to the left, while the characteristic peak near 22° almost stayed the same. This disparity in the d-spacing change revealed that the preferential binding of CB into the microporous regions with a greater chain spacing squeezed the surrounding polymer chains, thus leading to an increase in chain spacing and the formation of cavities with larger free volumes. The resulting higher free volume is conducive to increasing the gas permeation rate. In addition, the incorporation of CB formed more dedicated transport channels for smaller gases (H_2_ and CO_2_), thereby elevating their permeability and further enhancing the molecular sieving ability of the membranes. However, CB contents of 5 wt % or above results in two new peaks at 14.3° and 21.8°, corresponding to d-spacing of 6.23 and 4.15 Å, which are the characteristics of CB. At this point, the Matrimid chains are no longer able to completely pack around CB, and the polymer chain suffers from the extrusion of a large amount of CB around it, with the two interchain regions showing obvious contraction. The d-spacing of 5.99 and 4.09 Å shifted to 5.93 and 4.04 Å, respectively, implying that the increasing of CB content tightens the chain spacing and potentially enhances the membrane molecular sieving properties. To gain further insights into the effect of CB incorporation on the microstructures of the membrane, the pore size distribution (PSD) of membranes was characterized from the corresponding H_2_ adsorption isotherm using the nonlocal density functional theory method. The H_2_-derived Brunauer-Emmett-Teller (BET) surface areas were 0.54 to 0.77 and 0.56 m^2^/g for Matrimid, Matrimid-CB-3%, and Matrimid-CB-5%, respectively, indicating that CB effectively regulated the micropores of Matrimid-CB membrane. As shown in Fig. 3B and fig. S4, ultramicropores and micropores at 3.87 and 6.89 Å exist in the Matrimid membrane, and the size of the micropores increased from 6.89 to 8.49 Å with the loading of CB increasing from 0 to 3 wt %. With a further increasing of CB content from 3 to 5 wt %, the original micropores split into two pore sizes with dimensions of 7.07 and 9.08 Å, respectively, which are associated with the enhanced gas permeability. Within the MMMs, the pore sizes between polymer segments located in different regions around the three-dimensional (3D) supramolecular macrocycle are not always identical, which relates to the 3D conformation of the supramolecular complex and the distribution of the surrounding polymer chains (*52*). Both the carbonyl group at the CB end and the methylene groups on the side chains serve as hydrogen bonding sites (*53*). At low loadings (1 to 3 wt % CB loading), CB preferentially binds to Matrimid via stronger hydrogen bonding sites, resulting in a unimodal pore structure. With further increasing CB content, multisite hydrogen bonding forms between CB and Matrimid, and the micropore dimensions approximately correspond to the bottom spacing and surrounding spacing dimensions (5.80 and 9.10 Å, respectively) (*49*), thus resulting in a bimodal PSD centered at 7.07 and 9.08 Å. The incorporation of CB into the Matrimid membrane not only regulates the micropores size but also tailors the content of micropores and ultramicropores. A comparison between the Matrimid membrane and Matrimid-CB-3% membranes showed a notable increase in the content of micropores and ultramicropores (Fig. 3C), with the content of micropores and ultramicropores increasing by 428 and 323%, respectively, which could notably enhance the gas permeation selectivity. When the CB content was further elevated to 5 wt %, the content of ultramicroporous decreased by 63% compared to the Matrimid-CB-3% membrane, but the microporous content remained essentially unchanged, suggesting that the latter has a fewer number of accessible pores in the ultramicroporous range, which are associated with weakened gas selectivity. These results demonstrate that the microporous structure of the Matrimid-CB membranes can be notably modulated by adjusting the CB mass loading.

**Fig. 3.**
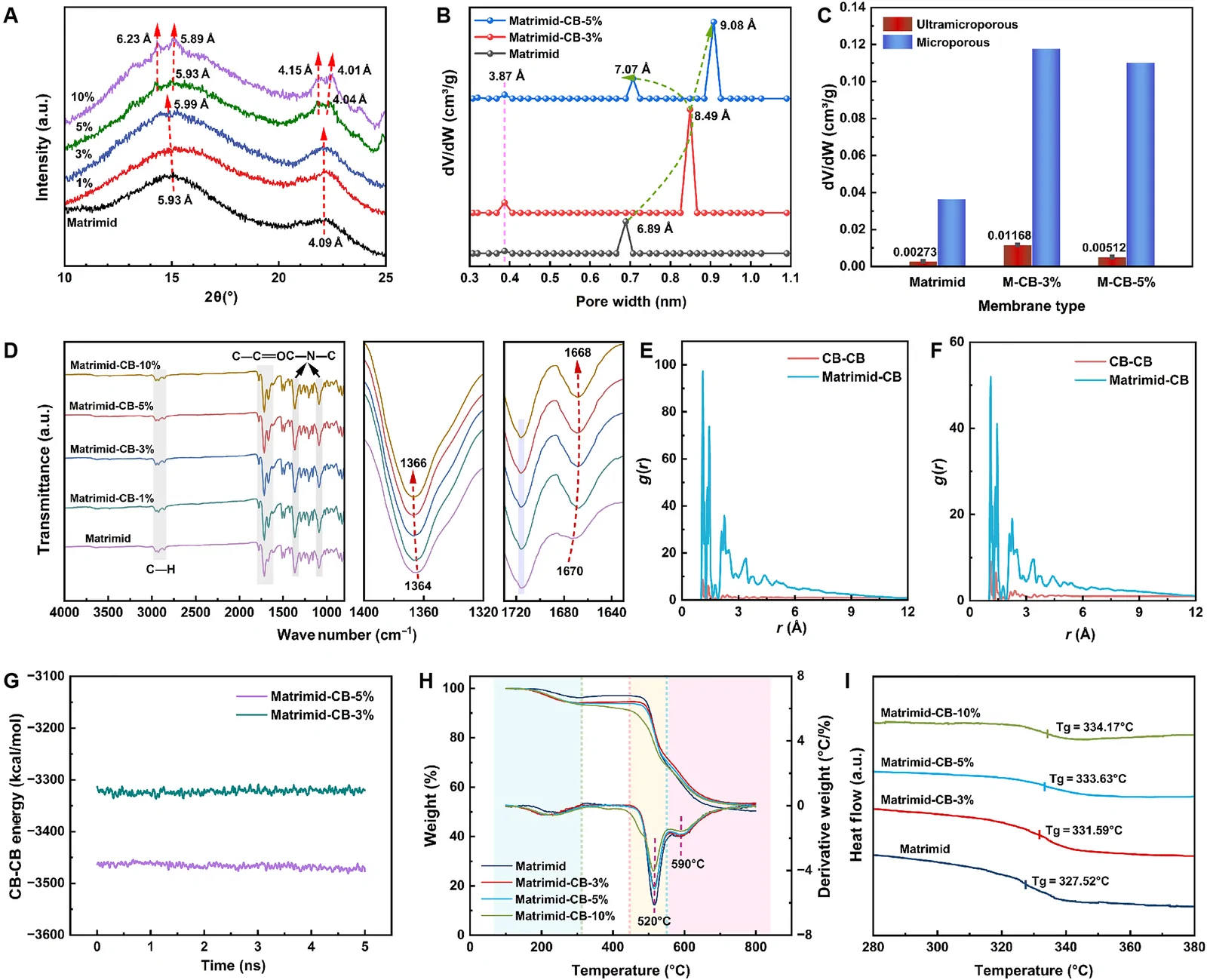
Structure characterization of Matrimid-CB membranes. (**A**) XRD patterns of the Matrimid-CB membranes. (**B** and **C**) PSD of the Matrimid-CB membranes (the PSD was calculated from the corresponding H_2_ adsorption isotherm using the DFT method). (**D**) ATR-FTIR spectra of the Matrimid-CB membranes. Radial distribution functions (RDFs) of Matrimid-CB and CB-CB correlations in the (**E**) Matrimid-CB-3% membrane and (**F**) Matrimid-CB-5% membrane. (**G**) Interaction energy between CB-CB in the Matrimid-CB membranes. (**H**) TGA plots of the Matrimid-CB membranes. (**I**) DSC plots of the Matrimid-CB membranes. a.u., arbitrary units.

The chemical structure of Matrimid-CB membranes was confirmed via Fourier transform infrared–attenuated total reflectance (FTIR-ATR) spectroscopy. As shown in Fig. 3D, the characteristic absorbance band at 1670 cm^−1^ corresponds to the symmetric stretching vibration of benzophenone carbonyl, the band at 1360 cm^−1^ corresponds to the stretching vibrations of C-N in the imide five-membered ring, and the bands at 2860 and 2890 cm^−1^ arise from the C-H stretching aliphatic. Compared with Matrimid membranes, the incorporation of CB resulted in a red shift of the characteristic C─C═O peak at 1670 cm^−1^ for all Matrimid-CB membranes, which was attributed to the formation of hydrogen bonding interaction between the benzophenone carbonyl group of Matrimid and CB. With the increasing of CB loading, the characteristic C-N peak at 1360 cm^−1^ of all Matrimid-CB membranes shows a blue shift, demonstrating a change in the corresponding chemical environment that may be attributed to hydrogen bonding between the five-membered imide ring carbonyl of Matrimid and CB. In contrast, the aliphatic C-H stretching bands remain largely unaffected, indicating minimal interaction at those sites. Overall, the hydrogen bonding interaction between Matrimid and CB is mainly established between the carbonyl group of Matrimid and the methylene group of CB. The absence of the characteristic peaks of CB in the MMMs (fig. S5) suggests the uniform dispersion of CB and its effective encapsulation within the Matrimid matrix. Hydrogen bond, a type of supramolecular interaction, generally manifests a peak in the radial distribution function (RDF) curve within the range of 0 to 3 Å (*54*). The RDF curves of the Matrimid-CB exhibit multiple pronounced peaks in this region, confirming the formation of hydrogen bonds between CB and Matrimid (Fig. 3, E and F). The RDF [*g*(*r*)] values between Matrimid and CB are substantially higher than those observed between CB molecules themselves, indicating stronger local interactions at the Matrimid-CB interface and comparatively weaker CB-CB interactions. As CB loading increased from 3 to 5 wt %, a notable reduction was observed in the maximum *g*(*r*) values between Matrimid and CB, while a slight increase was observed in the *g*(*r*) values between CB molecules. These findings demonstrate that an increase in CB loading results in a notable reduction in the local aggregation of Matrimid chains, while no substantial change was observed in the degree of aggregation among CB molecules, which confirms that the CB incorporated into the membrane is uniformly distributed among the polymer chains. To further elucidate the interfacial interactions, molecular dynamics (MD) simulations were conducted. As shown in Fig. 3G, the interactions between CB molecules are substantially enhanced with increasing CB loading from 3 to 5 wt %, indicating a more tightly packed and stable local arrangement of neighboring CB under higher loading conditions. CB molecules exhibit substantially stronger interactions with Matrimid than among themselves (fig. S6), promoting uniform dispersion within the polymer matrix. This reduced the possibility of nonselective pores forming due to the aggregation of CB, while the selective gas-transporting cavities provided by CB enhanced the gas transport efficiency for smaller gases and gas pair selectivity.

Thermogravimetric analysis (TGA) demonstrated that both Matrimid and Matrimid-CB membranes exhibited excellent thermal stability, retaining structural integrity up to 450°C (Fig. 3H). The mass loss that occurred between 120° and 300°C is attributed to the removal of the residual solvent from the membrane. There is a sharp weight decrease ranging from 450° to 550°C in the case of membranes, at which point the intense pyrolysis of the methyl side groups from Matrimid occurs. The final weight loss corresponds to the decomposition of the aromatic backbones, which begins at ∼550°C. With increasing CB loading, the glass transition temperature (*T*_g_) of the membranes increased from 327.5°C (Matrimid) to 334.2°C (Matrimid-CB membranes), which points toward the increased rigidity of the Matrimid chains around the Matrimid-CB interface and indicates the “wrapping” of the CB by the Matrimid chains (Fig. 3I). These results demonstrate that the interactions between Matrimid and CB enable the Matrimid chains to tightly encapsulate CB, facilitating the homogeneous dispersion of CB within the Matrimid-CB membranes. Notably, by adjusting the loading amount of uniformly dispersed CB in the membrane, the microporous structure of the membranes can be effectively modulated while simultaneously enhancing the structural stability.

### Gas transport mechanism of Matrimid-CB membranes

For gas separation membranes, changes in the micropore structure often have great impacts on gas transport properties. To investigate the mechanism by which changes in the micropore structure influence gas transport performance, the MD simulations were performed. Figure 4A shows the membrane structure model. This model was generated by using MD simulation based on the Large-scale Atomic/Molecular Massively Parallel Simulator (LAMMPS23) software together with the pysimm program within a periodic cubic cell with dimensions of 50 Å by 50 Å by 50 Å. The MD simulation generates a 3D membrane structure that closely matches the experimental PSD and density of membranes (Fig. 4E and table S1). Both H_2_ (kinetic diameters, 2.89 Å; radius, 1.45 Å) and N_2_ (kinetic diameters, 3.64 Å; radius, 1.82 Å) were used as probe molecules in the membrane model to analyze the pore spatial distribution, the interconnectivity of void spaces, the gas-accessible volume, and the average transmembrane path length. Figure 4B illustrates the spatial distribution of pores with radius sizes exceeding 1.2 Å. The incorporation of CB enhances the dispersion of pores and alters the content of different pore sizes, resulting in notable changes in the membrane structure. By contrast, the Matrimid-CB-5% membrane contained a higher prevalence of pores with a radius smaller than 1.45 Å, with 35% of pores being impassable to H_2_, which is consistent with the BET analysis results. This may be due to the spatially close CB molecules, which stack in a window-to-window dislocated manner (fig. S7). After solvent evaporation, this arrangement forms small channel-type structures that are impermeable even to H_2_ molecules (*53*). Figure 4C and fig. S8 present the interconnectivity of H_2_ with N_2_-accessible void space within membranes. Compared to the Matrimid membrane, the Matrimid-CB-3% and Matrimid-CB-5% membranes formed continuous linear and cluster-like channels with notably enhanced pore connectivity. Among them, the continuous H_2_-passable pore space length increased from 784 to 1004 Å (Fig. 4F). This confirms that CB with a cavity facilitates the formation of highly interconnected pore networks within Matrimid membranes. As shown in Fig. 4D and fig. S9, when H_2_ and N_2_ are used as probe gases for the membrane, some channels existing within the Matrimid-CB membranes selectively permit H_2_ transport while restricting N_2_. In addition, the fractional accessible volume for H_2_ (accessible volume: 14526 Å^3^) is greater than that for N_2_ (accessible volume: 11193 Å^3^) in the Matrimid-CB-3% membrane (Fig. 4G). This suggests that the membrane is able to accommodate more H_2_ than N_2_, facilitating its mass transfer. These results demonstrate that the presence of CB creates special transport channels for H_2_, increasing the transport space for H_2_ within the membrane and enhancing both the number and connectivity of gas transport channels. Consequently, this effectively improves the H_2_ transmission capacity and the sieving performance of gas pairs in the membrane.

**Fig. 4.**
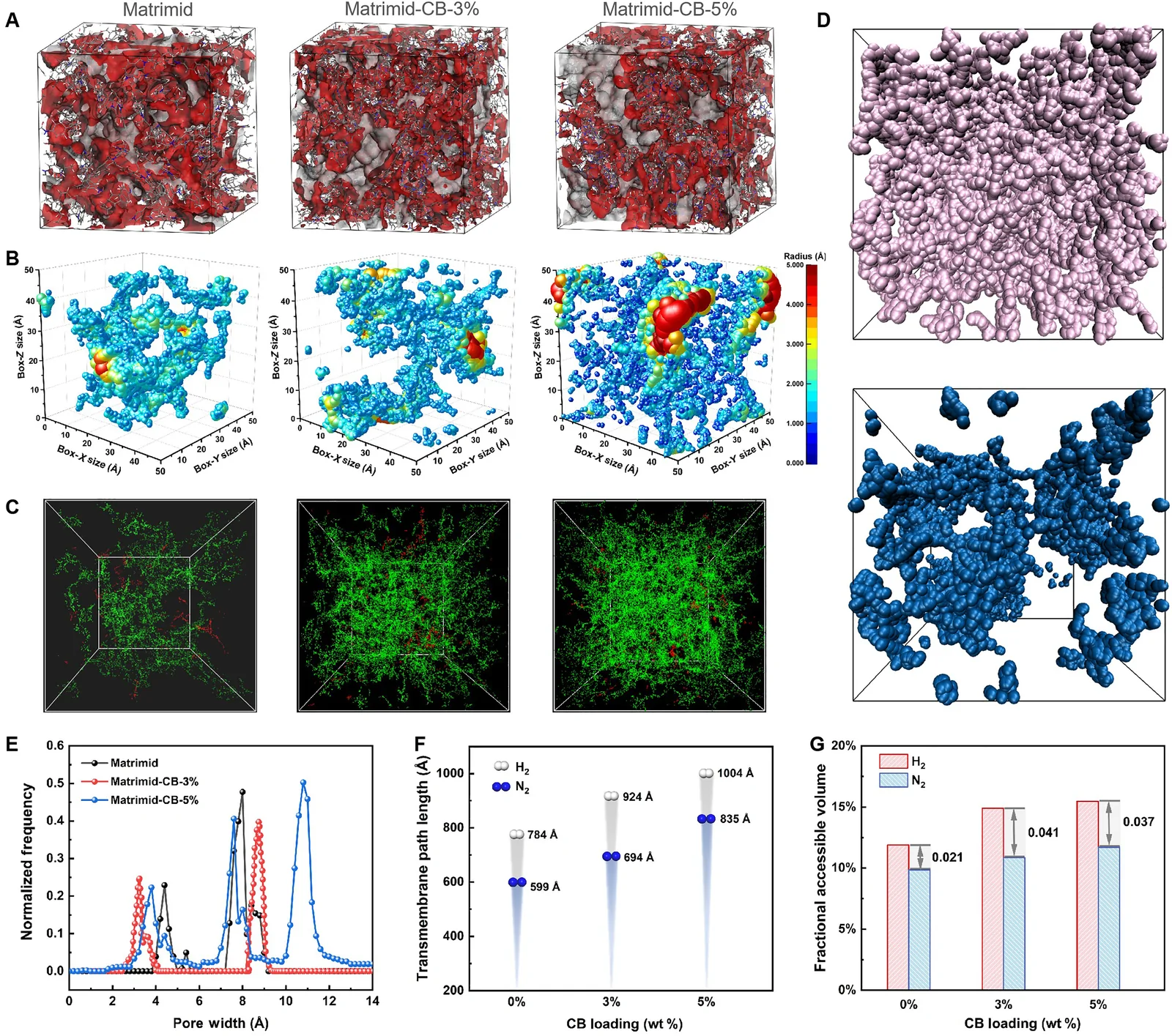
Structural analysis of Matrimid-CB-*n*% membrane models (*n*% is the CB mass loading, *n* = 0, 3, and 5). (**A**) 3D view of Matrimid-CB membrane models. The gray and red shades indicate that the free volume was detected by a probe with a radius of 1.45 Å. (**B**) The void distribution (with size distinguished by color) of Matrimid-CB membranes. Each color corresponds to the largest probe radius that could be inserted (for ease of observation, only the pores as detected by a probe with a radius of 1.20 Å are shown in the figure). (**C**) Interconnected (green) and disconnected (red) voids in the Matrimid-CB membrane models with respect to a probe of 1.45-Å radius. (**D**) Accessible void spaces for H_2_ (top) and N_2_ (bottom) in Matrimid-CB-3% membrane model. (**E**) Simulated PSD of the Matrimid-CB membranes. (**F**) The average continuous transmission path length for H_2_ and N_2_ was calculated on the basis of the accessible void space of the Matrimid-CB membranes. (**G**) The accessible fractions of free volume of H_2_ and N_2_ within the Matrimid-CB membranes were determined by probes with radii of 1.45 and 1.82 Å, respectively.

### Gas separation performance of Matrimid-CB membranes

To explore the gas molecular sieving performance in the Matrimid and Matrimid-CB membranes, the single gas permeation was conducted using different gas molecules including H_2_ (2.89 Å), CO_2_ (3.30 Å), N_2_ (3.64 Å), and CH_4_ (3.80 Å). The gas permeability and selectivity data of the membranes are described in table S2. Figure 5A presents the gas molecular size effect on gas permeability of Matrimid and Matrimid-CB membranes. All these membranes show the sharp cutoff between CO_2_ (3.30 Å) and N_2_ (3.64 Å) molecules, suggesting a clear gas molecular sieving capability. The introduction of CB enhances the molecular sieving effect with a promising separation potential for hydrogen purification, natural gas purification, and flue gas carbon capture. Although the larger gas molecules could penetrate the CB cavity owing to molecular rotation, they generally encounter a much higher energy barrier to pass through the windows (*55*). This contributes to the membrane’s precise molecular-sieving property. Upon introducing CB, the permeability to small gas molecules (e.g., H_2_ and CO_2_) increases markedly. The sudden jump of H_2_/N_2_ and H_2_/CH_4_ separation factors at 1 wt % loading suggests a percolation effect (Fig. 5, B and C). From this loading onward, small-molecule gases (H_2_ and CO_2_) are predominantly going through CB within the membrane, enhancing the gas transfer rate while substantially improving the gas pair selectivity (Fig. 5D). The enhancement of CB content (1 to 5 wt %) extends the fast, small-molecule gas-specific gas transport pathways provided by CB. Consequently, the permeability of small-molecule gases (i.e., H_2_ and CO_2_) increases notably when more CB is loaded. Simultaneously, the selectivity of several gases is slightly improved (Fig. 5, B and C). CB has an inner cavity with a large internal space, a small port size, and a height of 0.90 nm. This feature serves as a straight, fast gas transmission channel for small gas molecules. An increase in CB content enhances the selective channel network’s connectivity within the membrane, thereby reducing the tortuosity of small gas transmission paths and increasing transmission speed. As the CB content increases from 5 to 10 wt %, the gas permeabilities exhibit a visible increase, while H_2_/N_2_ and H_2_/CH_4_ selectivities slightly decrease. This may be attributed to the emergence of nonselective pores in the membrane structure. XRD analyses showed that characteristic peaks attributed to CB appeared in the membrane at CB contents of 5 wt % or above, indicating that the Matrimid chains were no longer able to completely encapsulate CB. The intermolecular forces formed between spatially close CB brought them closer together, and the CB molecules were stacked window-to-window in a misaligned manner to form a column, a structure that formed a channel-type structure after the loss of solvent molecules. However, the size of this channel is too small to allow all the gas molecules to penetrate, and the BET tests show that this channel size is even smaller than the MD diameter of H_2_. At the same time, these aggregated CB molecules influenced the stacking of polymer chains around them, increasing the d-spacing of polymer chains, thereby leading to a slight increase in permeability for all gases and weakening the molecular sieving capacity of the membrane.

**Fig. 5.**
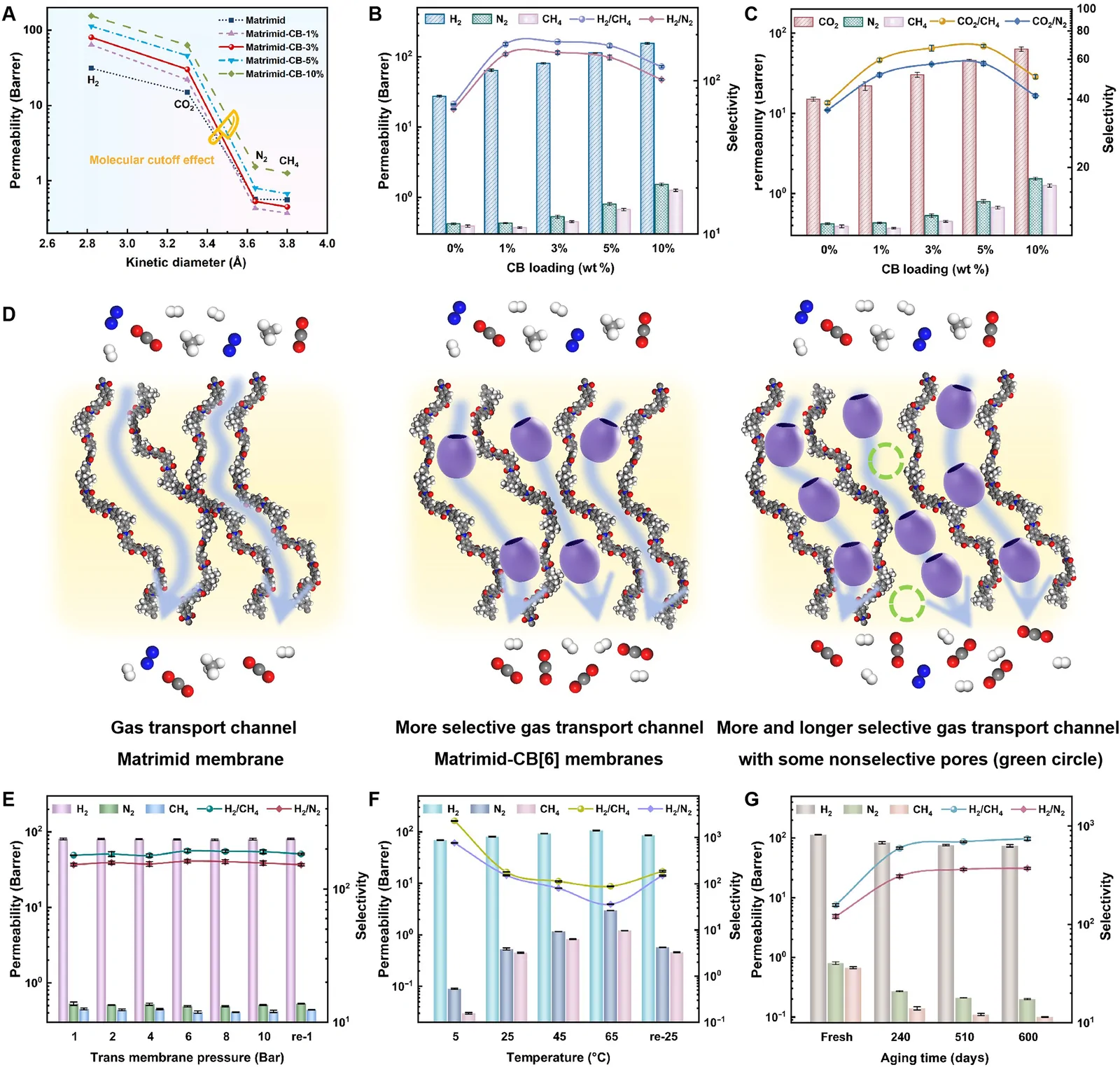
Gas separation performance of Matrimid-CB membranes. (**A**) Correlations between the permeabilities and molecular kinetic diameters of various gases. (**B** and **C**) Permeability and selectivity changes as a function of the CB mass loading. (**D**) Schematic comparison of gas transport through Matrimid-CB-*n*% membranes versus Matrimid membrane (*n*% is the CB mass loading, *n* = 1, 3, 5, and 10). (**E**) Effect of feed gas pressures on the separation performance of Matrimid-CB-3% membrane. (**F**) Effect of operating temperature on the separation performance of Matrimid-CB-3% membrane. (**G**) Long-term stability of the Matrimid-CB-5% membrane (the membrane was stored in an ambient environment without the introduction of any protective gas at room temperature).

To explore the stability and practical applicability of the membranes under harsh feed conditions, the membranes were tested at 25°C under different feed pressures (1 to 10 bar) using a gas permeation device, and the data were presented in table S3. The MMMs were stable at high pressures, at least up to 10 bar. As shown in Fig. 5E, the H_2_ permeability decreased slightly (∼1.03%) as the total feed pressure increased from 1 to 10 bar, whereas the H_2_/CH_4_ selectivity increased from 179.47 to 190.13 (a 6.04% increase), and the H_2_/N_2_ selectivity remained essentially unchanged.

The effect of operating temperature on the separation performance of the Matrimid-CB-3% membrane at 1 bar for H_2_/N_2_ and H_2_/CH_4_ was also investigated. For these two gas pairs (H_2_/N_2_ and H_2_/CH_4_), the permeability of all gases increased substantially, whereas the selectivity decreased gradually as the temperature increased from 25° to 65°C (Fig. 5F and table S4). After cooling down to 25°C, the performance could fully recover, indicating that the changes resulted from the intrinsic properties instead of membrane damage. Apparently, the membrane’s molecular sieving capacity for gas pairs diminished rapidly as the temperature increased. This is because the thermal motion of polymer chains becomes more intense at high temperatures, leading to an increase in the number of nonselective transient pores and accelerating the permeation rates of all gases. The permeability of N_2_ and CH_4_, gases with relatively low permeability, grew even more substantially, resulting in a deterioration in the membrane’s separation performance. In industrial applications, H_2_/CH_4_ selectivity >50 is needed for hydrogen recovery to meet process specifications (*34*). Notably, the Matrimid-CB-3% membrane achieved an H_2_/CH_4_ selectivity of 88° at 65°C and maintained a high screening capacity under elevated temperatures.

The aging behavior of Matrimid-CB membranes was evaluated by exposing them to the ambient environment without protective measures at room temperature for 600 days. Unexpectedly, upon aging, the separation performance of membrane improved markedly. However, as the aging time extended from 510 to 600 days, the changes in both gas permeability and selectivity became negligible, indicating that the gas transport performance of the membrane stabilized (Fig. 5G). The fresh Matrimid-CB-5% membranes exhibited lower separation selectivity for several industrially relevant gas pairs, including H_2_/N_2_, H_2_/CH_4_, CO_2_/N_2_, and CO_2_/CH_4_, compared to the Matrimid-CB-3% membrane. However, after aging, the separation selectivity increased to a greater extent in the Matrimid-CB-5% membrane compared to the Matrimid-CB-3% membrane (table S2). Physical aging commonly results in a reduction in excess free volume as the polymer chains densify into more compact packing. The changes in permeability and selectivity due to membrane aging typically follow the same trend as the performance variations observed in fresh membranes (*56*–*58*). After multiple repeated experiments, experimental errors were conclusively ruled out. The substantially different aging profiles of the Matrimid-CB-3% membrane and the Matrimid-CB-5% membrane prompted us to investigate the underlying reasons for the notably enhanced selectivity of the Matrimid-CB-5% membrane after aging.

The gas separation mechanism of membranes is typically investigated via gas adsorption testing, with gas solubility generally calculated from the gas adsorption capacity and gas diffusivity calculated on the basis of the solution-diffusion model (*59*). In our work, the solution-diffusion model is used solely as a phenomenological tool to extract the diffusion coefficient (*D*) from the gas permeability. Actually, the gas transport in the Matrimid-CB membrane can be described by the pore-flow mechanism more appropriately. As shown in fig. S10A, the solubility coefficients slightly decreased as the membranes aged, while the magnitude of the decrease in solubility selectivity was basically the same. This suggests that the total free volume of both membranes diminished during aging (*14*). The diffusion coefficients of the gases notably declined as aging progressed, whereas diffusion selectivity increased substantially (fig. S10B and table S5). This indicates that the evolution of membrane performance originates from the increased disparity in the diffusion coefficients of different gases within the membrane. As we mentioned in the section on gas transport mechanisms, the incorporation of CB regulates the microporous structure of the membrane, thereby substantially enhancing gas transport performance. The presence of CB-selective cavities increases the transport space for small gas molecules within the membrane, enhances pore connectivity, and enables faster transport with more pathways for small gas molecules, thereby improving permeability while substantially increasing selectivity. The aging-induced performance evolution of the Matrimid-CB membrane further supports the pore-flow transport mechanism. Specifically, gas molecules transport through channels formed by interconnected pore cavities within the membrane, connected by narrow “bottlenecks” (*60*). As the dimensions of pore cavities progressively decrease during membrane aging (*61*), the connections between pore cavities, or bottlenecks, also narrowed over time. The shrinkage in pore size leads to the constriction of accessible transport channels for gas molecules (*59*), thereby restricting gas transport within the membranes, and this effect is more pronounced for larger gas molecules. When gas molecules pass through narrow bottlenecks, the diffusion coefficient becomes highly sensitive to molecular size. Larger molecules (N_2_) encounter greater energy barriers and steric hindrance within narrow channels, resulting in a more substantial decrease in the diffusion coefficient, whereas smaller molecules (CO_2_) are less affected (*62*). Consequently, as the aging time progresses, the difference in diffusion coefficient between the two gases gradually increases, and the permselectivity of the gas pair gradually increases. Although some pore cavities are larger than the kinetic diameter of N_2_, the probability of N_2_ successfully traversing continuous pore channels is substantially lower than that of CO_2_, resulting in a gradual increase in gas permselectivity with aging time. Notably, the diffusion selectivity of the Matrimid-CB-5% membrane exceeded that of the Matrimid-CB-3% membrane after aging. This suggests that increasing the CB content within the membrane enhances the disparity in diffusion inhibition experienced by CO_2_ and N_2_ in aged membranes. The stable selective gas transport cavity structure of CB effectively mitigates the negative effects of pore shrinkage during long-term aging, maintaining the permeability of small gas molecule transport pathways. Consequently, Matrimid-CB-5% membranes exhibit higher gas separation capabilities after aging.

When depicted on permeability-selectivity trade-off plots, the Matrimid-CB membranes already surpassed the 2008 Robeson upper bound from 1 wt % loading onward for H_2_/N_2_ (Fig. 6A) and even from 3 wt % onward for H_2_/CH_4_ (Fig. 6B). The data were provided in table S6. The gas separation performance outperforming most existing Matrimid-based membranes reported in the literature is attributable to both the unique properties of the CB filler and the optimized membrane morphology. The H_2_ permeability of the Matrimid-CB-3% membrane achieved up to 81 Barrer, with H_2_/CH_4_ separation selectivity reaching up to 180. This performance notably exceeds those of widely used commercial materials such as Matrimid, cellulose acetate, and polysulfone. In particular, the membrane exhibited excellent anti-aging performance. After 600 days of aging, the H_2_ permeability of Matrimid-CB-5% membrane decreased from 113 to 74 Barrer, a reduction of 35%, while the separation selectivities for H_2_/N_2_ and H_2_/CH_4_ reached up to 372 and 743, respectively, representing improvements of 3-fold and 4.7-fold over the fresh Matrimid-CB-5% membrane, respectively. In addition, the Matrimid-CB-5% membrane also exhibits excellent performance for other crucial gas pairs including CO_2_/N_2_ and CO_2_/CH_4_ (Fig. 6, C and D, and table S7). The gas separation performance of the Matrimid-CB-5% membrane after 600 days of aging also exceeds the latest gas separation upper bound line, outperforming most Matrimid-based membranes and other MMMs. From an application perspective, these membranes, offering high selectivity and superior anti-aging performance, are promising candidates for hydrogen and natural gas purification, with the potential to reduce capital costs, minimize membrane replacement frequency, and enable the obtainment of high-purity clean energy.

**Fig. 6.**
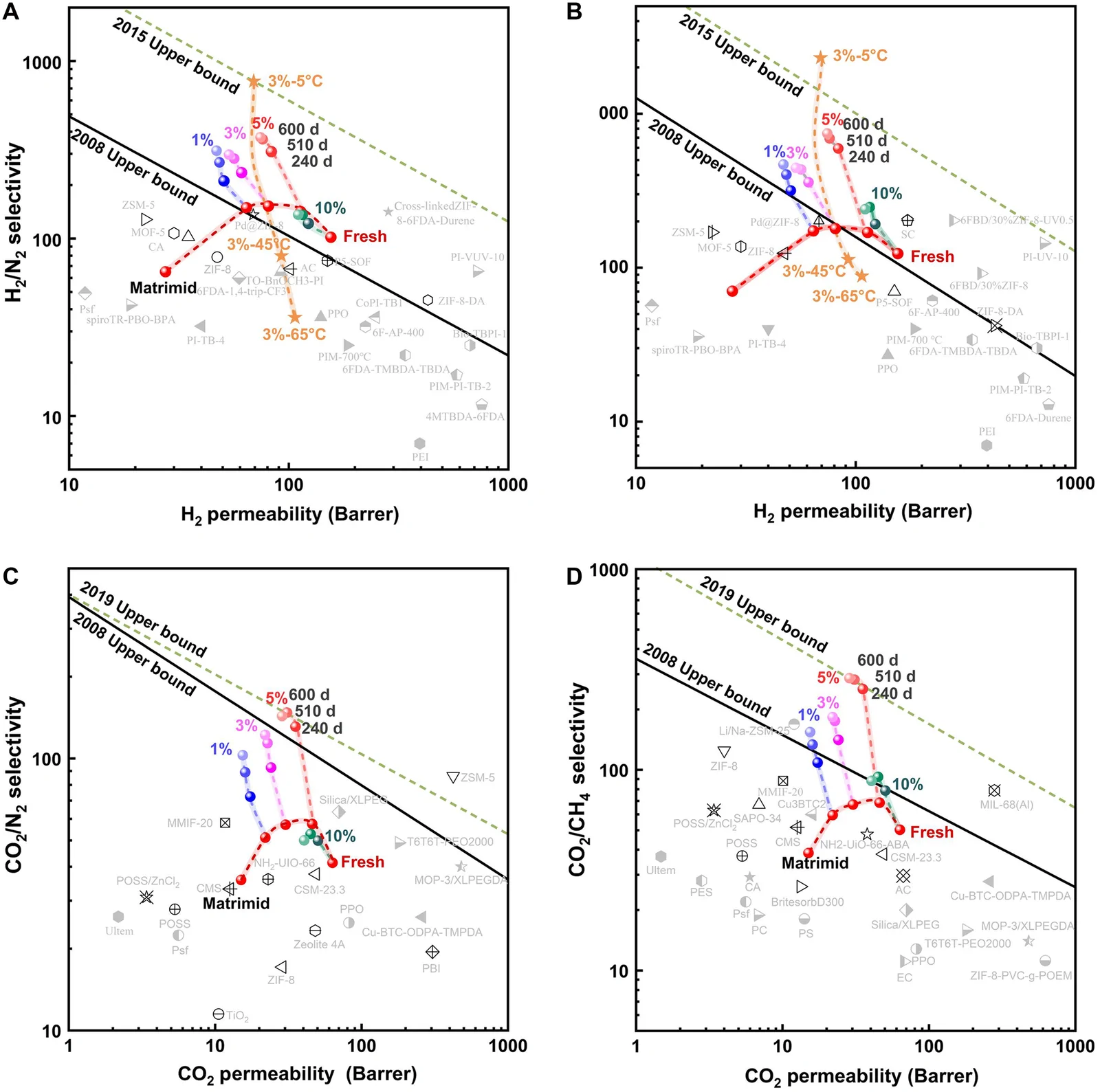
Gas separation performance of Matrimid-CB membranes compared with other polymeric membranes. Plots of (**A**) H_2_/N_2_, (**B**) H_2_/CH_4_, (**C**) CO_2_/N_2_, and (**D**) CO_2_/CH_4_ separation performance. (The red symbols are gas data of fresh membranes, while the other symbols are gas data of membranes after physical aging, the black symbols are Matrimid-based membranes, and the gray symbols are other polymer-based membranes. Lines are drawn to guide eyes.) d, days.

## DISCUSSION

In summary, the incorporation of CB into the Matrimid polymer matrix as a selective gas transport cavity has conclusively demonstrated its ability to tailor microporous and ultramicroporous structures within the membrane. The hydrogen bonding interactions between CB and Matrimid enhanced their interfacial compatibility, enabling uniform dispersion of CB within the membrane. In addition, the integration of CB enhanced the connectivity of gas transport network among the polymer chains, which is beneficial for the permeability and selectivity improvement. The resultant membrane exhibited exceptional separation performance for H_2_/N_2_ and H_2_/CH_4_, exceeding the 2008 Robeson upper bound line. After 600 days of aging test, the H_2_/CH_4_ selectivity of Matrimid-CB-5% membrane increased from 169 to 743, while the H_2_ permeability decreased from 113 to 74 Barrer, with only 35% loss. The rigid cavity of CB played a pivotal role in maintaining the structural stability and patency of the small gas transport channels they created, conferring excellent anti-aging performance during long-term service. These results demonstrate that Matrimid-CB membranes with cavity-shaped molecular sieving channels have great potential for H_2_ purification and CO_2_ capture from flue or natural gas.

## MATERIALS AND METHODS

### Materials

CB (>97%, C_36_H_36_N_24_O_12_) from Shanghai BeiDi Pharmaceutical Technology Co. was supplied as a fine solid powder. Matrimid was supplied by Huntsman Advanced Materials. ReagentPlus-grade *N*,*N*-dimethylformamide (DMF) solvent (≥99.5%) purchased from China National Pharmaceutical Group was used directly for making polymer solutions without further purification. Matrimid 5218 was dried under vacuum at 120°C for 12 hours before use. Methanol solvent (≥99.5%) from China National Pharmaceutical Group was directly used as received. High-purity H_2_ gas (99.999%) and CH_4_ gas (99.9%) were supplied in compressed gas cylinders from Nanjing Nanyuan Gas Company with limited liability, CO_2_ gas (99.9%) was supplied by Nanjing Shangyuan Industrial Gas Plant, and N_2_ gas (99.999%) was supplied by Nanjing Special Gas Plant.

### Fabrication of pristine Matrimid membrane

A raw Matrimid dope solution was prepared by dissolving pure Matrimid powder (0.2 g) in DMF (8.0 g) solvent, stirred for 10 hours, and sonicated for 2 hours. The clean Matrimid dope solution was obtained by filtering the raw Matrimid dope solution through a 1.2-μm polytetrafluoroetylene (PTFE) syringe. The clean dope solution was then directly poured into a clean petri dish by a syringe and placed in a vacuum oven to heat from room temperature to 120°C for 8 hours to evaporate the solvent. The original membrane, which still contained a small amount of DMF solvent, was peeled off, and a 14-mm-diameter circular membrane was cut from the original membrane, which was submerged in methanol for 12 hours to remove the residual DMF from the membrane by solvent exchange. Then, the membranes were placed in a petri dish and dried in a vacuum oven at 120°C for 8 hours to obtain membrane samples that could be tested directly with a thickness of 50 ± 5 μm.

### Fabrication of the Matrimid-CB MMMs

MMMs containing 1, 3, 5, and 10 wt % CB (in Matrimid) were prepared. First, a raw Matrimid dope solution was prepared by dissolving 0.2 g of pure Matrimid powder in 8.0 g of DMF solvent, stirred for 10 hours, and sonicated for 2 hours. The clean Matrimid dope solution was obtained by filtering the raw Matrimid dope solution through a 1.2-μm PTFE syringe. Then, CB powder (2.0, 6.0, 10.0, or 20.0 mg) was added to 5.0 g of DMF solvent, and the mixture was stirred for 10 hours and sonicated for 2 hours to obtain the CB dispersion. Half of the polymer solution was mixed with the CB dispersion, stirred on a heatable magnetic stirring plate for 10 hours, and sonicated twice (1 hour per turn) at the beginning and at the end of the stirring process. Last, the remaining polymer solution was added and stirred at 80°C, and the concentration of the mixed solution was adjusted to be ∼2 to 3 wt % to obtain a solution concentration similar to that of the pristine membrane. The mixture was then injected mixture into a clean petri dish by a syringe and was then transferred into the 120°C vacuum oven at ambient temperature for 8 hours of solvent evaporation. The residual DMF was similarly removed from the Matrimid-CB membranes by solvent exchange with methanol. These CB-incorporated MMMs had an average thickness of 50 ± 5 μm.

### Membrane characterization

The surface and cross-sectional morphologies of the pristine Matrimid and Matrimid-CB-*n*% membranes were observed by SEM (GeminiSEM 450). The chain packing structure and d-spacing of the Matrimid and Matrimid-CB-n% membranes were examined by a Bruker D8 Advance x-ray diffractometer with the x-ray radiation source being Cu K- (1.54 Å) and the diffraction angles ranged from 10° to 25° with a scan speed of 5°/min. The average d-spacing was evaluated on the basis of the Bragg’s law (*n*λ = 2*d*sinθ), where *n* is an integer (1, 2, 3), λ represents the wavelength of the x-ray radiation source, *d* means the average interchain/intersegmental distance between adjacent polymer chains in semicrystalline regions (d-spacing), and θ denotes the XRD angle. FTIR spectroscopy (Bruker TENSOR II) was used to analyze the chemical structures of pristine Matrimid and Matrimid-CB-*n*% membranes in ATR mode with the spectra over the wave number range of 800 to 4000 cm^−1^. FTIR spectroscopy (Bruker TENSOR II) was used to analyze the chemical structures of CB powders with the spectra over the wave number range of 500 to 4000 cm^−1^. Thermal stability of the pristine Matrimid and Matrimid-CB-*n*% membranes was measured by a thermogravimetric analyzer (Q5000IR) in the temperature range of 25° to 800°C with a heating rate of 10°C/min under a flowing N_2_ atmosphere (50 ml min^−1^). Differential scanning calorimetry (DSC) was performed on a calorimeter (TA Instruments DSC Q2000) in the temperature range of 50° to 800°C with a heating rate of 10°C/min under a flowing N_2_ atmosphere (100 ml min^−1^). The BET (Quantachrome Autosorb IQ) technique was used to measure the pore structure of the prepared membrane. The membrane has been freeze-dried for 10 hours before degassing. Membrane samples were degassed under a high vacuum at 120°C for 8 hours before analysis.

### MD simulation

MD simulation was performed using the LAMMPS23 software together with the pysimm program (*63*). Initial amorphous cells were generated using the PACKMOL package (*64*) by randomly packing the molecular building units into a periodic simulation box. To represent the polymeric membrane morphology, the polymer structures were not treated as simple stacked monomers; instead, reaction-based bond formation was carried out in pysimm to construct polymer chains/crosslinked networks by connecting predefined reactive sites according to a distance criterion until the targeted conversion/degree of crosslinking was reached. After each reaction step, the structure was relaxed via energy minimization and short MD relaxation to remove local strain (*65*, *66*). The loading of CB was set to 0, 3, and 5 wt %, consistent with the experimental compositions. Periodic boundary conditions were applied in all three dimensions. The optimized potentials for liquid simulations-all atom (OPLS-AA) force field was used to describe bonded and nonbonded interactions. Lennard-Jones and short-range Coulomb interactions were computed with a cutoff distance of 12 Å, while long-range electrostatics were treated using the particle-particle particle-mesh (PPPM) method.

The molecular force field consists of nonbonded and bonded interactions. The nonbonded interaction contains van der Waals (vdW) and electrostatic interactions, which are described by Eqs. 1 and 2, respectively

$$E_{\mathrm{LJ}}\left(r_{\mathrm{ij}}\right)=4\varepsilon_{\mathrm{ij}}\left[{\left(\frac{\sigma_{\mathrm{ij}}}{r_{\mathrm{ij}}}\right)}^{12}-{\left(\frac{\sigma_{\mathrm{ij}}}{r_{\mathrm{ij}}}\right)}^{6}\right]$$ (1)

$$E_{c}\left(r_{\mathrm{ij}}\right)=\frac{q_{i}q_{j}}{4\pi \varepsilon_{o}\varepsilon_{r}r_{\mathrm{ij}}}$$ (2)

In these equations, $\sigma_{\mathrm{ij}}$ is the atomic diameter, ε is the atomic energy parameter, $\varepsilon_{o}$ is the vacuum permittivity, $\varepsilon_{r}$ is the relative permittivity of the medium, $\varepsilon_{\mathrm{ij}}$ is the depth of the potential well. $E_{\mathrm{LJ}}\left(r_{\mathrm{ij}}\right)$ is the potential energy between two particles as a function of the distance $r_{\mathrm{ij}}$ between them. $E_{c}\left(r_{\mathrm{ij}}\right)$ is the potential energy between two charged particles as a function of the distance. $r_{\mathrm{ij}}$ between them. $q_{i}$ and $q_{j}$ are atomic charges, and $r_{\mathrm{ij}}$ is the distance between atoms.

For different atom types, the vdW interactions were calculated using geometric mixing rules, as given by Eqs. 3 and 4

$$\sigma_{\mathrm{ij}}=\frac{1}{2}\left(\sigma_{\mathrm{ii}}+\sigma_{\mathrm{jj}}\right)$$ (3)

$$\varepsilon_{\mathrm{ij}}={\left(\varepsilon_{\mathrm{ii}}*\varepsilon_{\mathrm{jj}}\right)}^{\frac{1}{2}}$$ (4)

Before the MD simulation, all models were first energy-minimized by using the steepest-descent algorithm and then equilibrated under the number of particles, pressure, temperature (NPT) ensemble to achieve stable density and relaxed packing. In detail, the system was first annealed under the NPT (*P* = 101 kPa) ensemble for a total time of 2000 ps at cycling temperatures ranging from 300 to 500 K. The MD simulation of the system was performed under the number of particles, volume, temperature (NVT) ensemble (*T* = 300 K) for 5000 ps to obtain the equilibrated structures. The temperature and pressure were controlled by the Nose-Hoover thermostat and the Parrinello-Rahman barostat, respectively. The spatial density distributions of reaction atoms within the monomer molecules were also calculated. Structural visualization and image rendering were performed using OVITO software (*67*). The PSD was calculated using Zeo++ by analyzing multiple equilibrated configurations extracted from the MD trajectories (*68*). A total of *N* = 10,000 snapshots were sampled from the equilibrated portion of the trajectory and used for statistical averaging. PSD was computed using a probe insertion approach with H_2_ and N_2_ as probe molecules, using probe radii based on their kinetic diameters (H_2_: 2.89 Å, radius 1.45 Å; N_2_: 3.64 Å, radius 1.82 Å). In Zeo++, the PSD bin width was set to 0.1 Å, and 2000 Monte Carlo insertions were performed per snapshot. The reported PSD corresponds to the average over all snapshots.

### Gas permeation tests of membranes

Gas permeation properties of the membranes were measured using a variable-pressure constant-volume gas permeation cell. The dense membrane was mounted onto the permeation cell and vacuumed at 25°C for 12 hours before the gas permeation test was carried out. Gas permeability was tested following the order of H_2_, CO_2_, N_2_, and CH_4_ corresponding to the order of gas kinetic diameter (H_2_: 2.89 Å, CO_2_: 3.30 Å, N_2_: 3.64 Å, and CH_4_: 3.80 Å) from smallest to largest where the pressure was 1 bar. The cell temperature was kept constant at 25°C. The gas permeability coefficient through the membrane was calculated according to the steady-state pressure increment (*dp*/*dt*) via the following Eq. 5

$$P=\frac{273\times 10^{10}}{760}\frac{\text{Vl}}{\text{AT}\left(p_{2}\times \frac{76}{14.7}\right)}\left(\frac{\text{dp}}{\text{dt}}\right)$$ (5)

where *P* denotes the gas permeability coefficient in Barrer [1 Barrer = 1 × 10^−10^ cm^3^ (STP) cm cm^−2^ s^−1^ cmHg^−1^], *V* refers to the volume of the downstream reservoir (cubic centimeters), *A* is the effective membrane area (square centimeters), *l* represents the membrane thickness (centimeters), *T* is the testing temperature (kelvin), and $p_{2}$ is defined as the upstream pressure of the system (atm). The permeability tests were repeated at least three times, and the average deviation obtained was less than 5%.

The ideal selectivity between two different gases across a polymeric membrane is the ratio of their single gas permeability coefficient as described in the following Eq. 6

$$\alpha_{A/B}=\frac{P_{A}}{P_{B}}$$ (6)

where $P_{A}$ and $P_{B}$ refer to the permeability coefficients of gases A and B, respectively.

### Solubility and diffusion coefficient test

The barometric sorption apparatus was custom-made on the basis of the previously reported design by Koros and Paul (*69*). The test was conducted at 25°C. First, 0.5 g of membrane material was placed in the sample chamber, and the entire system was under vacuum conditions for 24 hours. Then, N_2_ was introduced into the system sequentially at pressures ranging from 1 to 12 bar. After completing the N_2_ test, the membrane sample was degassed for 12 hours, after which CO_2_ was loaded into the system. The gas adsorption capacity of the membrane at equilibrium was calculated using the pressure difference values obtained from two pressure sensors. Each membrane sample was tested three times, with an N_2_ test deviation of less than 10% and a CO_2_ deviation of around 5%. The obtained sorption isotherms of membranes were fitted to the dual-mode sorption model (*70*) as shown below (Eq. 7)

$$C=k_{D}p+\frac{C_{H}^{\prime }\text{bp}}{1+\text{bp}}$$ (7)

where *C*, *p*, *k*_D_, *C*’_H_, and *b* represent the gas concentration [cm^3^ (STP)/cm^3^ membrane], the feed pressure (bar), the Henry’s law coefficient [cm^3^ (STP)/cm^3^ membrane bar], the Langmuir capacity parameter [cm^3^ (STP)/cm^3^ membrane], and affinity constant (bar^−1^), respectively.

The solubility coefficient (*S*, unit: 10^−2^ cm^3^ cm^−3^ cmHg^−1^) for testing gas was calculated according to Eq. 8

$$S=\frac{C}{p}$$ (8)

where *p* is the feed pressure (bar), and *C* is the concentration of adsorbed testing gas [cm^3^ (STP)/cm^3^ membrane].

The solubility coefficient selectivity between two different gases across a polymeric membrane is the ratio of their single gas solubility coefficient as described in the following Eq. 9

$$\alpha_{S}=\frac{S_{A}}{S_{B}}$$ (9)

where $S_{A}$ and $S_{B}$ refer to the solubility coefficients of gases A and B, respectively.

The diffusivity coefficient (*D*, unit: 10^−8^ cm^2^ s^−1^) for testing gas could be calculated to Eq. 10

P=D×S (10)

The diffusion coefficient selectivity between two different gases across a polymeric membrane is the ratio of their single gas diffusion coefficient as described in the following Eq. 11

$$\alpha_{D}=\frac{D_{A}}{D_{B}}$$ (11)

where $D_{A}$ and $D_{B}$ refer to the diffusion coefficients of gases A and B, respectively.

## Supplementary Materials

### This PDF file includes:

Figs. S1 to S10 Tables S1 to S7 References
