# Supplementary material for: Regulating the microporous structure by cavity-shaped molecular sieving channels
Source: Sci Adv. 2026 Jul 29;12(31):eaee9782. doi: 10.1126/sciadv.aee9782 (PMC13418740; doi:10.1126/sciadv.aee9782)
Supplement: Supplementary file 1 [file sciadv.aee9782_sm.pdf]

Science Advances
AAAS

Supplementary Materials for

Regulating the microporous structure by cavity-shaped molecular sieving channels

Wen He *et al.*

Corresponding author: Jiangtao Liu, jiangtaoliu@ustc.edu.cn

Sci. Adv. **12**, eaee9782 (2026)
DOI: 10.1126/sciadv.aee9782

This PDF file includes:

Figs. S1 to S10
Tables S1 to S7
References

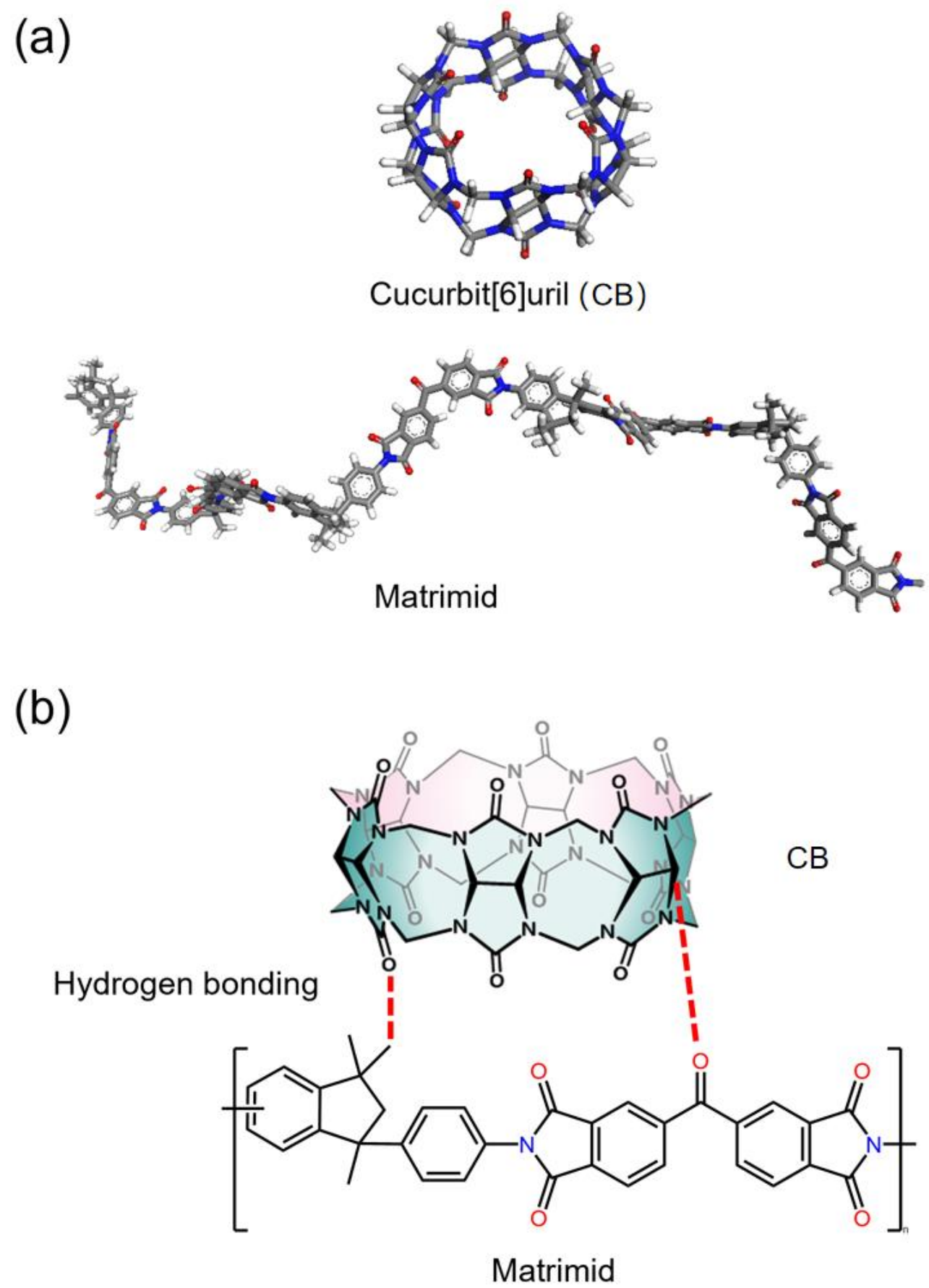

Fig. S1. Chemical structure of Matrimid and Cucurbituril (CB). (a) Chemical structures of Matrimid and CB. (b) Illustration of the interactions between CB and Matrimid polymer chains.

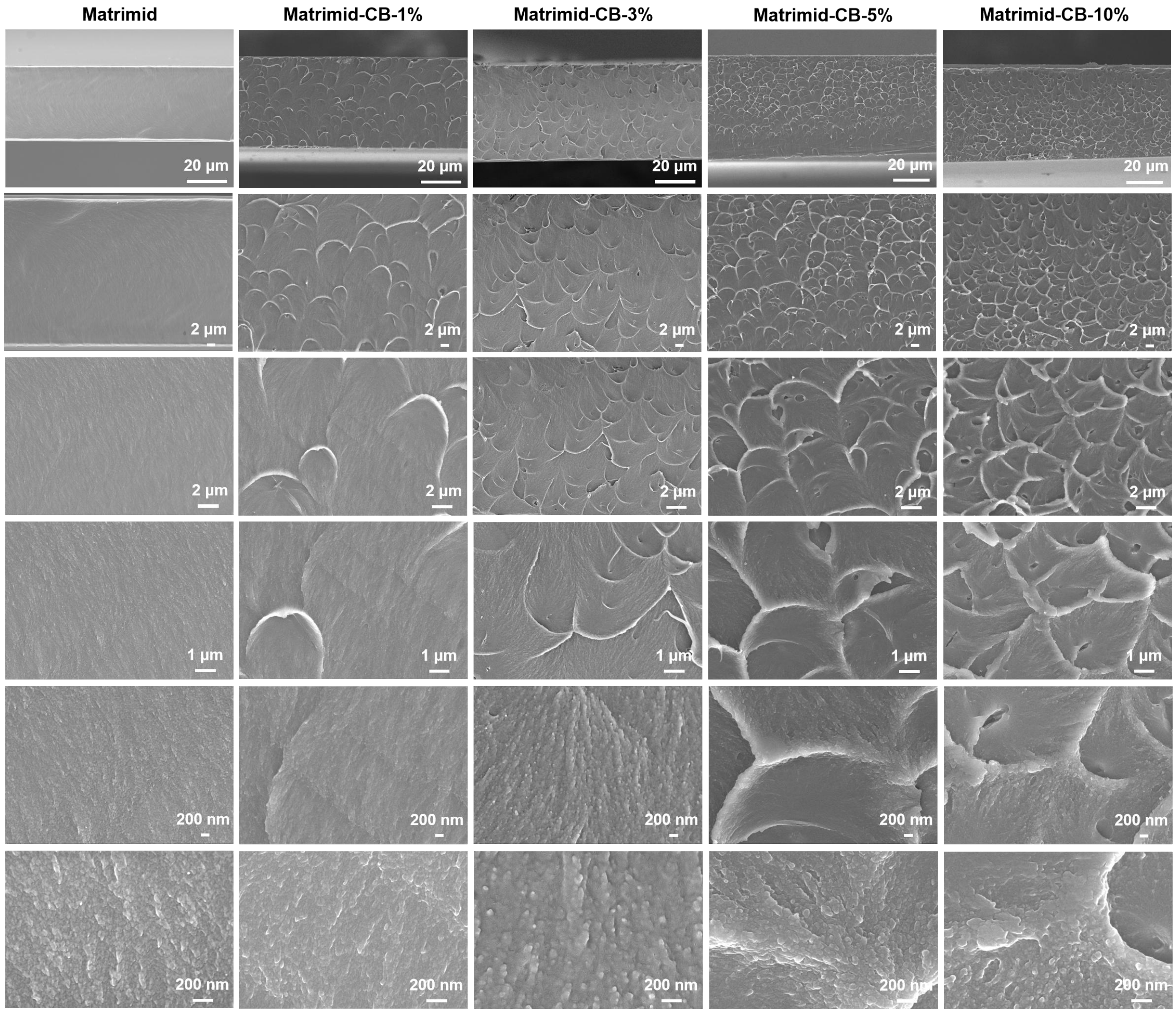

Fig. S2. SEM images of the cross section of Matrimid-CB-n% membranes up to ×50,000 magnification (n% is the CB mass loading and was varied from 0 to 10 wt%, n = 0, 1, 3, 5, 10). For n = 1, 3, 5, and 10, the 20 μm and 200 nm images are reused from Fig. 2B and 2C.

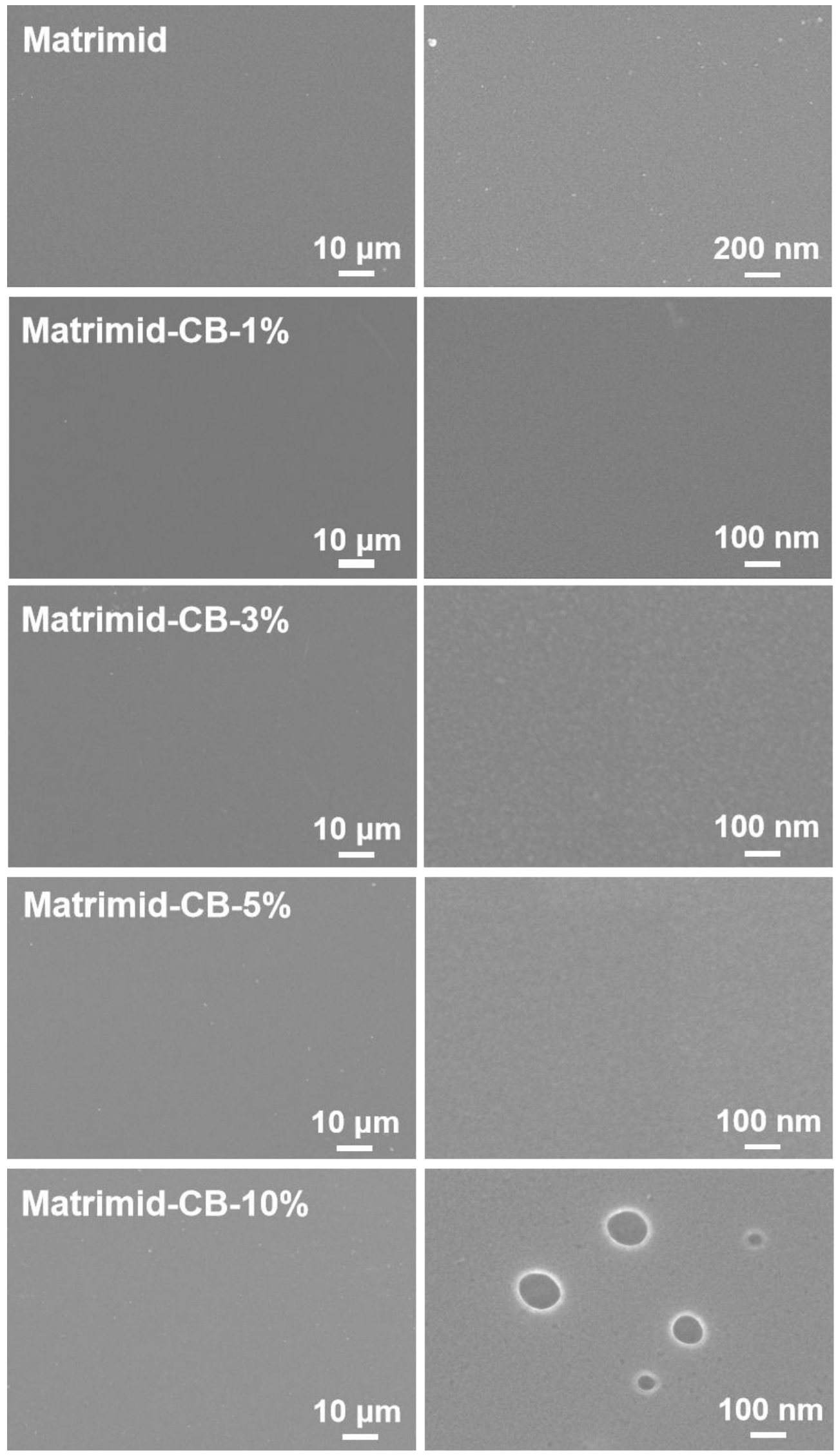

Fig. S3. SEM images of the surface section of Matrimid-CB-n% membranes up to ×50,000 magnification (n% is the CB mass loading and was varied from 0 to 10 wt%, n = 0, 1, 3, 5, 10).

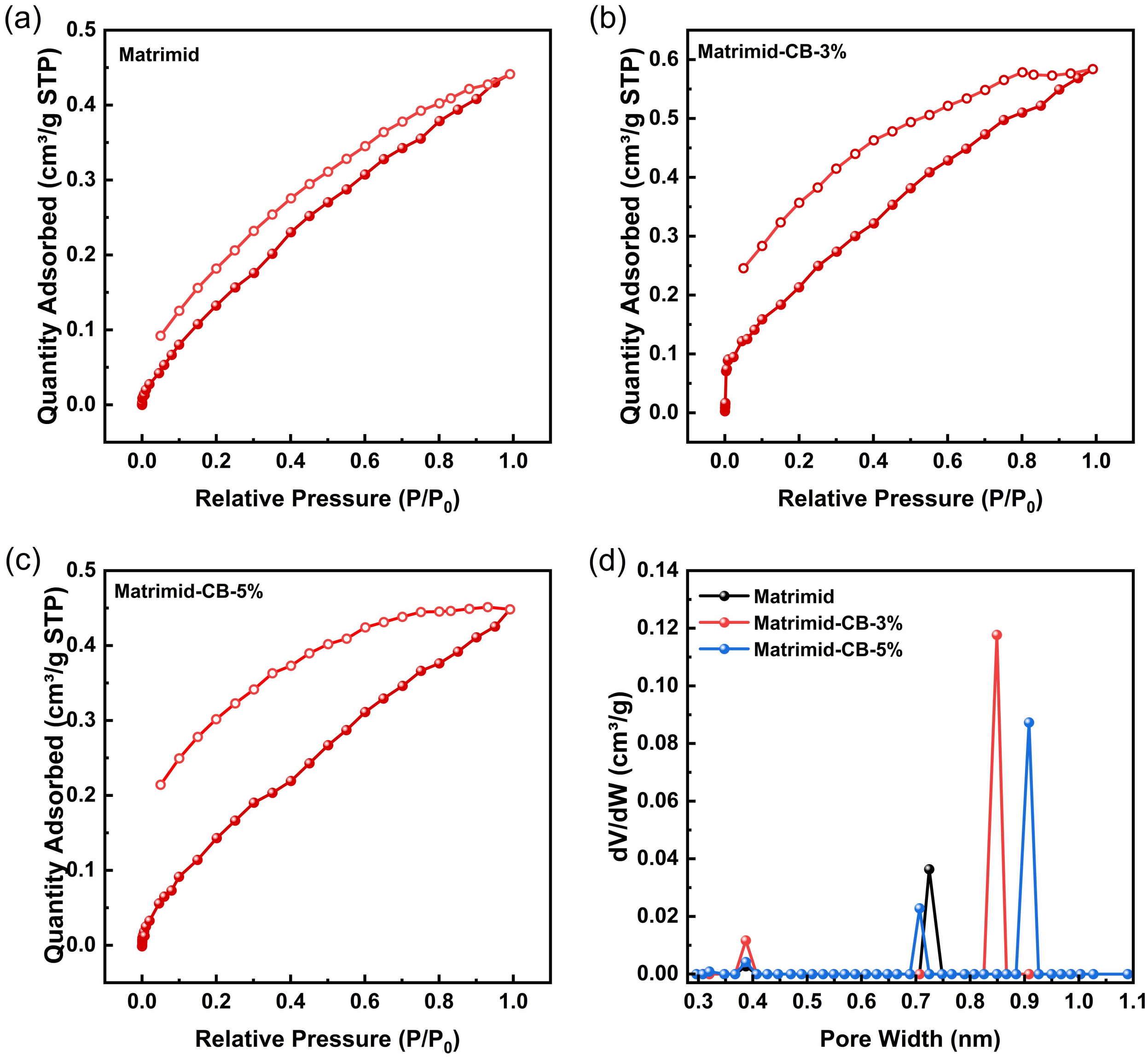

Fig. S4. BET surface area (a-c) and pore size distribution (d) of the Matrimid-CB membranes. The pore size distribution was calculated from the corresponding H_2 adsorption isotherm using the DFT method.

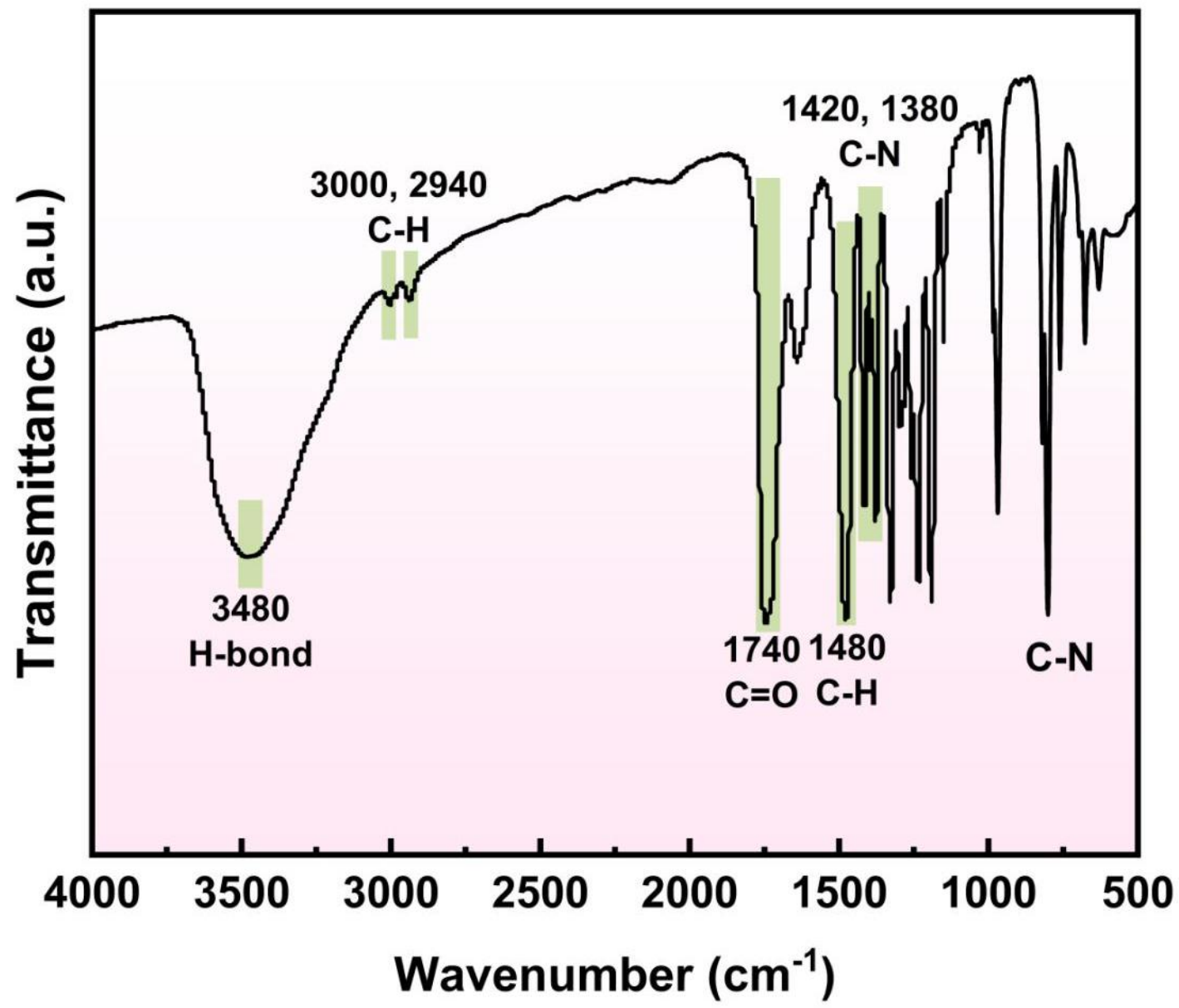

Fig. S5. Fourier-transform infrared (FTIR) spectra of CB powders.

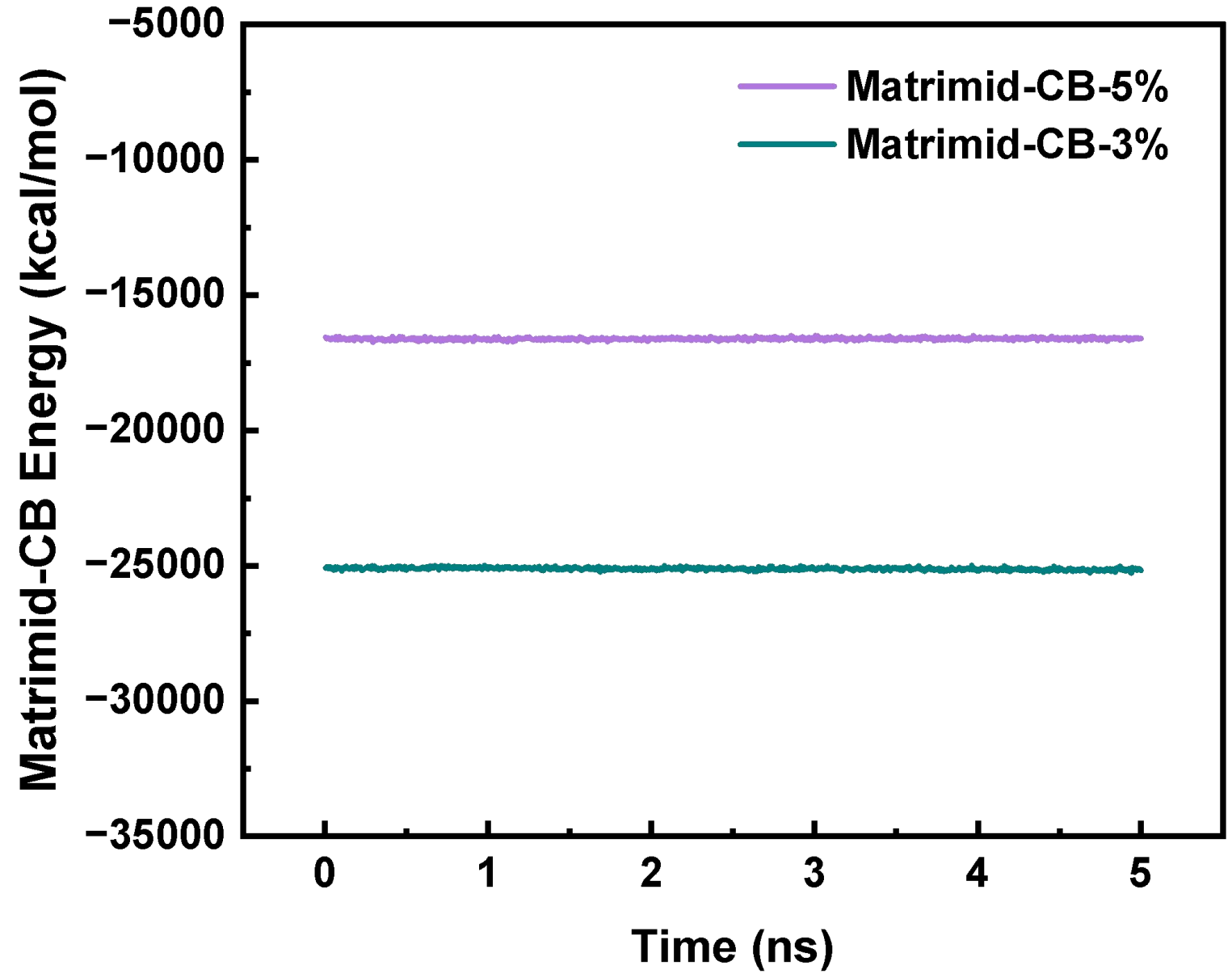

Fig. S6. Interaction energy between Matrimid and CB in the Matrimid-CB membranes.

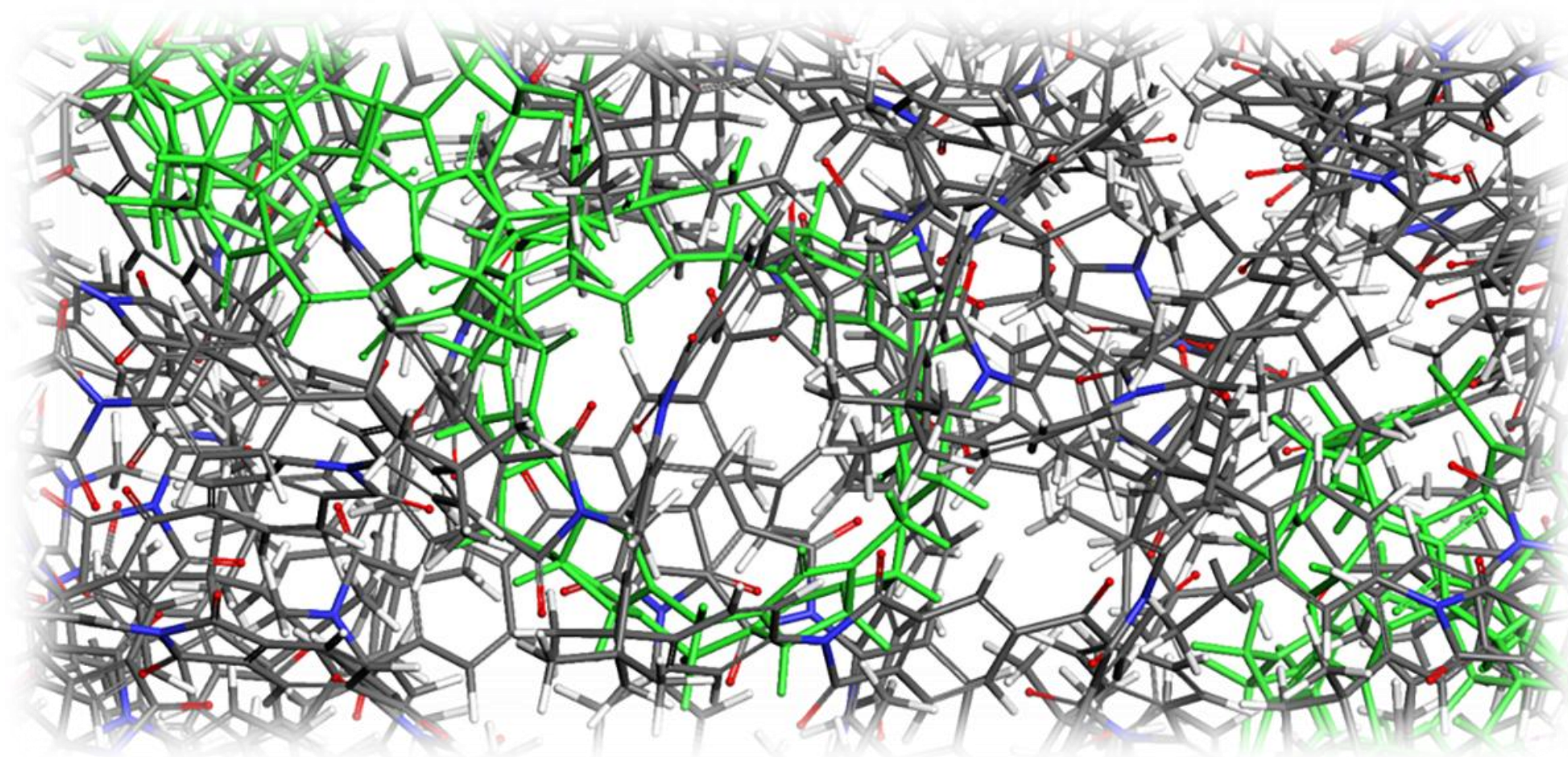

Fig. S7. The CB stack details of Matrimid-CB-5% membrane. The whole system is shown in a stick model (green structure: CB molecules).

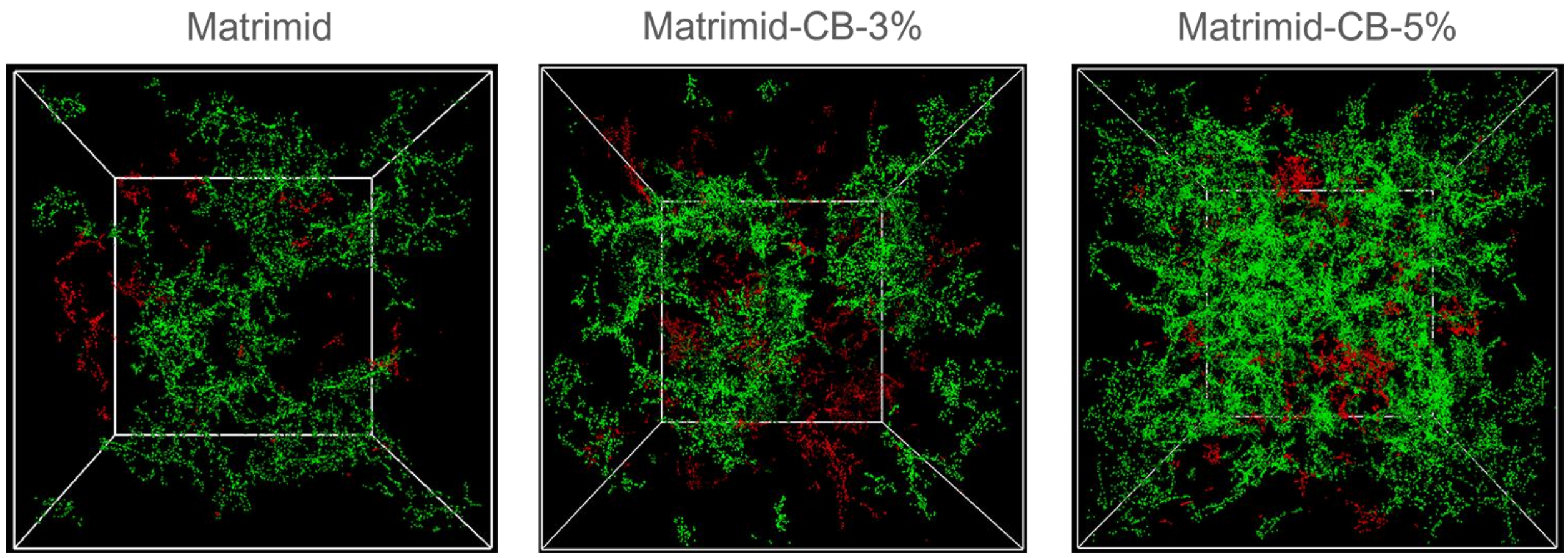

Fig. S8. Interconnected (green) and disconnected (red) voids in the Matrimid-CB membrane models with respect to a probe of 1.82-Å radius.

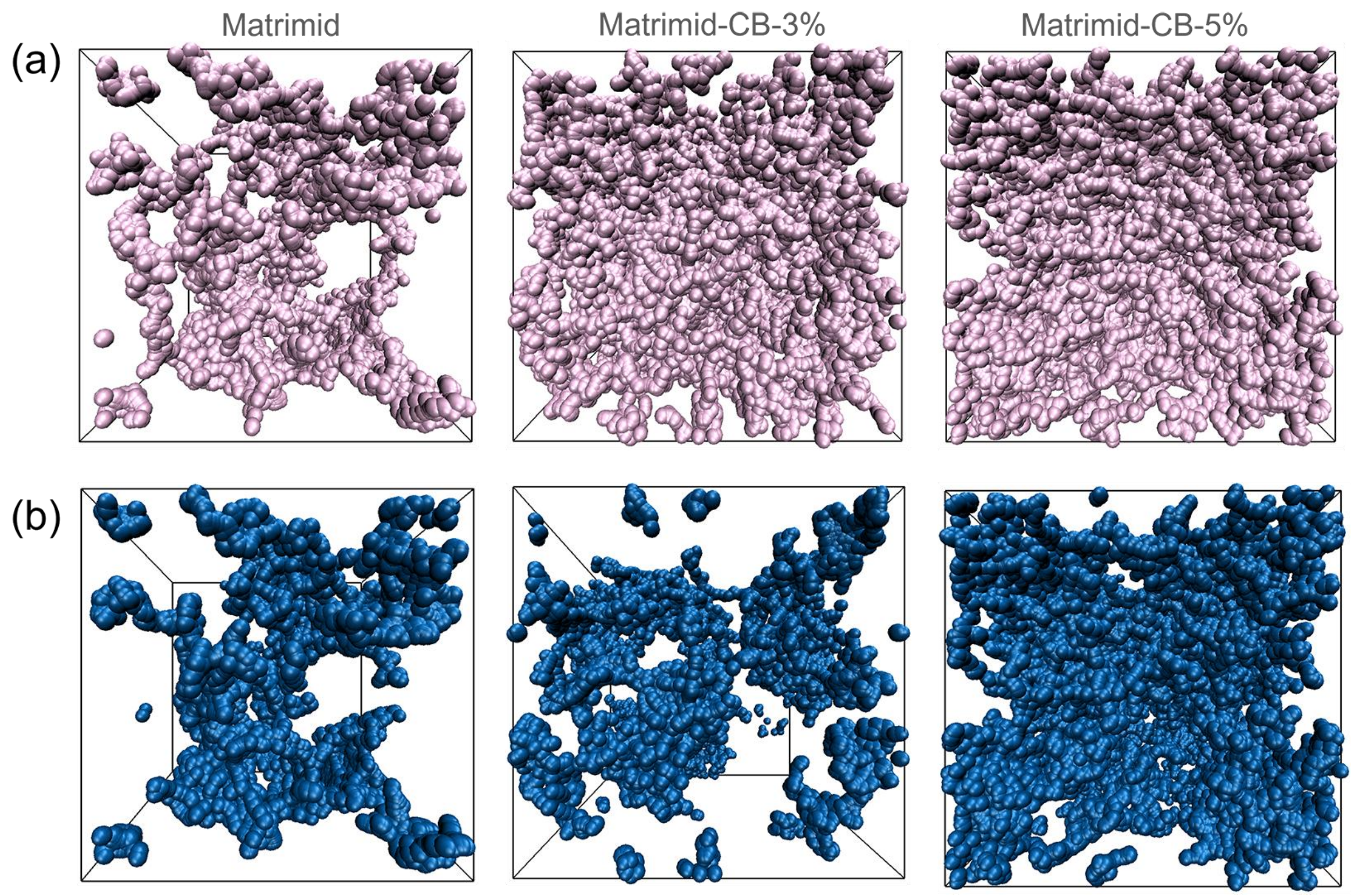

Fig. S9. Accessible void spaces for H_2 (a) and N_2 (b) in Matrimid-CB-n% membranes model (n% is the CB mass loading and was varied from 0 to 5 wt%, n = 0, 3, 5)**.**

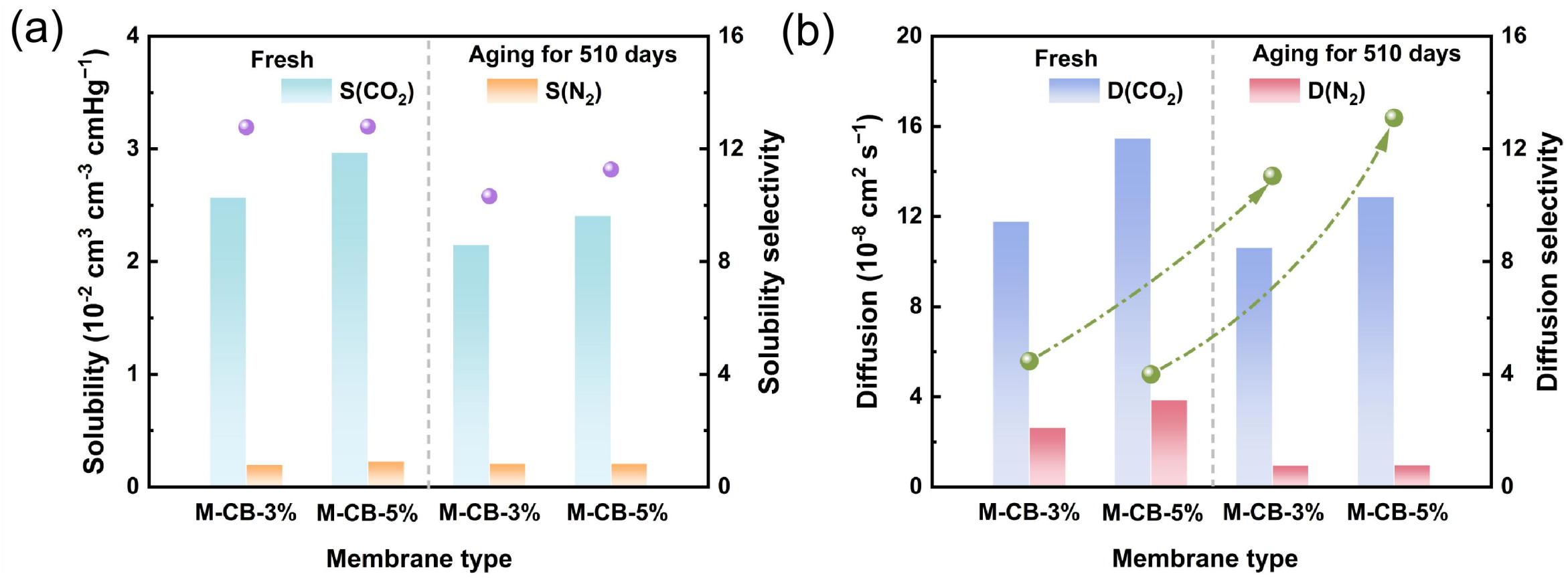

Fig. S10. (a) Solubility coefficients and solubility selectivity, (b) Diffusion coefficients and diffusion selectivity of CO_2 and N_2 for the Matrimid-CB-3% membrane, Matrimid-CB-5% membrane, Matrimid-CB-3% age membrane and Matrimid-CB-3% age membrane.

Table S1. The Density, Occupied Volume and Free Volume of Matrimid-CB-n% membranes derived from simulation (n = 0-5 indicated the degree of CB mass loading)**.**

Membrane	Density (g/cm^3)	Occupied Volume (Å^3)	Accessible Volume (Å^3)	
			H_2	N_2
Matrimid	1.13	44801.14	5324.75	5141.94
Matrimid-CB-3%	1.16	97514.72	14525.88	11192.92
Matrimid-CB-5%	1.15	100433.22	15544.15	13150.52

Table S2. Summary of gas transport properties of the Matrimid and Matrimid-CB-n% membranes at 1.0 atm and 25 °C (n = 1-10 indicated the degree of CB mass loading)**.**

Membrane		Permeability (Barrer)		Selectivity			
		H_2	CO_2	H_2/N_2	H_2/CH_4	CO_2/N_2	CO_2/CH_4
Fresh	Matrimid	27.45±0.85	15.03±0.32	65.00±0.37	70.38±0.53	35.79±0.18	38.54±0.67
	Matrimid-CB-1%	64.16±2.73	22.00±0.88	149.21±3.57	173.41±3.63	51.19±1.10	59.50±1.10
	Matrimid-CB-3%	80.76±2.11	30.25±1.75	152.38±3.79	179.47± 1.10	57.10±0.40	67.20±2.10
	Matrimid-CB-5%	113.41±1.20	45.99±1.20	141.76±5.43	169.27±4.80	57.49±1.31	68.64±0.88
	Matrimid-CB-10%	155.51±4.07	63.32±1.47	101.64±1.60	123.42±2.17	41.39±0.81	50.25±1.07
Aging for 240 days	Matrimid	20.92±0.34	12.39±0.23	67.48±2.19	74.71±0.40	39.97±1.19	44.25±0.37
	Matrimid-CB-1%	50.68±1.98	17.38±0.46	211.17±4.72	316.75±1.51	72.42±0.71	108.63±1.88
	Matrimid-CB-3%	61.97±1.45	24.09±1.24	238.35±0.85	364.53±6.38	92.65±2.28	141.71±6.46
	Matrimid-CB-5%	88.27±3.66	35.37±0.96	326.93±9.94	630.50±14.44	131.00±2.10	252.64±9.43
	Matrimid-CB-10%	122.83±2.04	50.42±2.01	121.61±2.33	191.92±3.12	49.92±0.22	78.78±0.56
Aging for 510 days	Matrimid	18.33±0.21	10.48±0.40	70.50±1.63	83.32±0.56	40.31±0.19	47.64±1.01

	Matrimid-CB-1%	48.32±2.00	16.03±0.31	268.44±8.15	402.67±9.98	89.06±2.36	133.58±2.79
	Matrimid-CB-3%	56.42±1.55	22.79±0.89	282.10±0.74	434.00±8.16	113.95±1.02	175.31±1.27
	Matrimid-CB-5%	81.07±2.32	30.99±1.18	386.05±9.23	737.00±12.40	147.57±4.90	281.73±2.12
	Matrimid-CB-10%	115.64±3.86	44.89±1.84	136.05±1.02	240.92±0.50	52.81±0.79	92.52±0.76
Aging for 600 days	Matrimid	18.06±0.12	10.22±0.17	72.24±0.98	82.09±2.46	40.88±0.26	46.46±1.06
	Matrimid-CB-1%	46.87±1.76	15.43±0.56	312.47 ± 4.96	468.70±3.53	102.87±1.88	154.30±0.81
	Matrimid-CB-3%	53.44±1.14	21.98±0.92	296.89±19.12	445.33±24.07	122.11±7.15	183.17±6.10
	Matrimid-CB-5%	74.32±3.47	28.66±1.23	371.60 ± 9.93	743.20±27.29	143.30±3.29	286.60±9.44
	Matrimid-CB-10%	110.42±2.97	40.52±0.64	136.32 ± 0.64	240.04±0.84	50.02±0.33	88.09±1.31

Table S3. Summary of gas transport permeability and selectivity of the Matrimid-CB-3% membrane at different pressures and 25 °C.

Pressure (Bar)	Gas permeability (Barrer)	Selectivity	
	H_2	H_2/N_2	H_2/CH_4
1	80.76±2.11	152.38±3.79	179.47±1.10
2	80.43±1.44	157.71±4.20	182.80±7.82
4	80.22±1.28	154.27±6.37	178.27±5.07
6	79.32±1.32	161.88±4.79	193.46±6.03
8	78.65±1.63	160.51±5.75	191.83±4.51
10	79.93±2.04	156.73±5.68	190.31±6.02
Return to 1	80.68±1.47	152.23±3.43	183.36±2.58

Table S4. Summary of gas transport permeability and selectivity of the Matrimid-CB-3% membrane at 1.0 atm and different temperatures.

Temperature (°C)	Gas permeability (Barrer)	Selectivity	
	H_2	H_2/N_2	H_2/CH_4
5	69.22±1.09	769.11±9.82	2307.33±40.67
25	80.76±2.11	152.38±3.79	179.47±1.10
45	92.58±1.43	79.81±0.68	112.90±4.11
65	106.67±2.24	35.92±0.49	88.16±1.42
Return to 25	85.84±1.64	150.60±4.58	186.61±7.78

Table S5. Solubility coefficients, diffusion coefficients, sorption selectivity and diffusion selectivity of Matrimid-CB-n%-Fresh membranes and Matrimid-CB-n%-Aging for 510 days membranes (n = 3-5 indicated the degree of CB mass loading)**.**

Membrane	CO_2		N_2		CO_2/N_2 Selectivity	
	S	D	S	D	α_S	α_D
Matrimid-CB-3%-Fresh	2.57	11.78	0.20	2.64	12.77	4.47
Matrimid-CB-5%-Fresh	2.97	15.48	0.23	3.87	12.79	4.00
Matrimid-CB-3%-Aging for 510 days	2.14	10.63	0.21	0.96	10.19	11.04
Matrimid-CB-5%-Aging for 510 days	2.21	14.02	0.21	0.98	10.52	14.31

Table S6. Summary of literature H_2/N_2 and H_2/CH_4 separation performance.

Membrane Name	H_2 Permeability (Barrer)	H_2/N_2 Selectivity	H_2/CH_4 Selectivity	Operating conditions	Gas Type	Reference
Matrimid-SC-3%	172.20	153.75	202.59	1.0 atm, 35 °C	Pure	26
Matrimid-MOF-5	30.00	107.10	137.00	2.0 atm, 35 °C	Pure	71
Matrimid-AC	101.00	67.30	95.80	1.0 atm, 30 °C	Pure	72
Matrimid-ZSM-5	22.23	127.90	169.80	1.5 atm, 35 °C	Pure	73
Matrimid-ZIF-8	47.20	78.60	124.00	2.0 atm, 35 °C	Pure	74
Matrimid-Pd@ZIF-8	68.85	135.90	201.10	5.0 atm, 25 °C	Pure	75
Matrimid-ZIF-8-DA	430.00	45.00	42.00	3.5 atm, 35 °C	Pure	76
Matrimid-CA	34.87	102.00	117.80	2.7 atm, 35 °C	Pure	77
Matrimid-P5-SOF	150.00	75.00	70.00	1.0 atm, 20 °C	Pure	78
PI-TB-4	40.00	32.10	40.00	1.0 atm, 35 °C	Pure	79
6F-AP-400	223.40	31.90	61.00	6.8 atm, 35 °C	Pure	80
6FDA-TMBDA-TBDA	339.00	21.00	34.00	--, 35 °C	Pure	81
Bio-TBPI-1	669.00	25.00	30.00	--, 35 °C	Pure	82
PIM-PI-TB-2	582.00	17.00	19.00	2.0 atm, 35 °C	Pure	83
CoPI-TB-1	249.00	36.00	36.00	1.0 atm, 35 °C	Pure	84
PI-VUV-10	720.00	65.50	144.00	1.0 atm, 35 °C	Pure	85
5TP-FDA-Durene	861.60	17.50	22.70	6.8 atm, 30 °C	Pure	86
cross-linked ZIF-8/6FDA-durene	284.00	141.40	203.30	3.5 atm, 35 °C	Pure	87
TO-BnOCH$_3$-PI	91.90	64.70	129.40	20 atm, 35 °C	Pure	88
6FDA-1,4-trip-CF3	59.40	60.00	112.10	8.8 atm, 35 °C	Pure	89
spiroTR-PBO-BPA	19.00	42.20	35.90	1.0 atm, 35 °C	Pure	90

Psf	11.80	49.20	53.60	4.4 atm, 35 °C	Pure	91
PEI	75.80	--	16.50	1.5 atm, 25 °C	Pure	92
PPO	110.00	--	36.00	2.0 atm, 25 °C	Pure	93
6FBD/30%ZIF-8	373.00	--	91.70	4.0 atm, 35 °C	Pure	94
6FBD/30%ZIF-8-UV0.5	278.00	--	203.00	4.0 atm, 35 °C	Pure	94
Matrimid	27.45	65.00	70.38	1.0 atm, 25 °C	Pure	This work
Matrimid-CB-1%	64.16	149.21	173.41	1.0 atm, 25 °C	Pure	This work
Matrimid-CB-3%	80.76	152.38	179.47	1.0 atm, 25 °C	Pure	This work
Matrimid-CB-5%	113.41	141.76	169.27	1.0 atm, 25 °C	Pure	This work
Matrimid-CB-10%	155.51	101.64	123.42	1.0 atm, 25 °C	Pure	This work

Table S7. Summary of literature CO_2/N_2 and CO_2/CH_4 separation performance.

Membrane Name	CO_2 Permeability (Barrer)	CO_2/N_2 Selectivity	CO_2/CH_4 Selectivity	Operating conditions	Gas Type	Reference
Matrimid-MOF-5	20.20	40.40	44.70	2.0 atm, 35 °C	Pure	71
Matrimid-AC	66.70	23.80	29.64	1.0 atm, 30 °C	Pure	72
Matrimid-ZIF-8	4.70	26.20	124.90	2.6 atm, 35 °C	Pure	74
Matrimid-POSS	5.30	53.00	37.20	10.0 atm, 35 °C	Pure	95
Matrimid-POSS+0.3M$ZnCl_2$	3.40	30.90	62.80	10.0 atm, 35 °C	Pure	95
Matrimid-CMS	12.60	33.20	51.70	3.5 atm, 35 °C	Pure	96
Matrimid-NH_2-UiO-66 ABA	37.90	--	47.70	9.0 atm, 35 °C	Mixed-50:50	97
Matrimid-MIL-68(Al)	279.60	--	82.20	1.0 atm, 100 °C	Pure	98
Matrimid-Britesorb D300	13.30	--	26.20	12.0 atm, 25 °C	Pure	99
Matrimid-MMIF-20	10.10	--	88.00	4.0 atm, 35 °C	Mixed-50:50	99
Matrimid-MMIF-20	11.70	58.00	--	4.0 atm, 35 °C	Mixed-50:50	100
Matrimid-SAPO-34	6.90	--	67.00	10.0 atm, 25 °C	Pure	100
Matrimid-Cu3(BTC)2	16.00	--	61.00	5.0 atm, 35 °C	Pure	101
Matrimid-CSM-23.3	48.60	37.60	38.00	9 atm, 35 °C	Mixed-50:50	102
Matrimid-TiO_2	10.50	11.50	13.70	2.0 atm, 35 °C	Pure	103
Matrimid-Zeolite 4A	48.30	23.40	--	2.0 atm, 25 °C	Pure	104
Matrimid-ZSM-5	423.00	86.20	--	10.0 atm, 25 °C	Pure	105
Matrimid-ZIF-8-30	28.70	17.10	24.90	4.0 atm, 22 °C	Pure	106
Matrimid-UIO-66-NH_2	23.00	36.00	--	10.0 atm, 25 °C	Pure	107
Matrimid-PBI	305.50	19.45	52.30	3.5 atm, 35 °C	Pure	108
MOP-3/XLPEGDA50	480.00	40.00	14.00	7.8 atm, 35 °C	Pure	109

T6T6T-PEO2000	180.00	49.00	16.00	4.0 atm, 35 °C	Pure	110
Silica/XLPEG	73.10	64.10	20.90	6.0 atm, 35 °C	Pure	111
Ethyl cellulose	67.70	21.30	11.10	2.0 atm, 25 °C	Pure	112
PPO	82.00	24.90	12.80	--, 35 °C	Pure	113
Polystyrene	14.10	28.80	18.10	4.4 atm, 23 °C	Pure	114
Polycarbonate	6.80	21.30	18.90	10 atm, 35 °C	Pure	115
Polysulfone	5.60	22.40	22.00	10.0 atm, 35 °C	Pure	116
Cellulose acetate	5.96	25.80	29.20	--, 35 °C	Pure	117
Ultem	1.48	27.40	37.00	3.5 atm, 35 °C	Pure	118
ZIF-8-PVC-g-POEM	623.00	--	11.20	1.0 atm, 35 °C	Pure	119
Cu-BTC-ODPA-TMPDA	260.70	26.10	27.76	2.0 atm, 35 °C	Pure	120
Matrimid	15.03	35.79	38.54	1.0 atm, 25 °C	Pure	This work
Matrimid-CB-1%	22.00	51.19	59.50	1.0 atm, 25 °C	Pure	This work
Matrimid-CB-3%	30.25	57.10	67.20	1.0 atm, 25 °C	Pure	This work
Matrimid-CB-5%	45.99	57.49	68.64	1.0 atm, 25 °C	Pure	This work
Matrimid-CB-10%	63.32	41.39	50.25	1.0 atm, 25 °C	Pure	This work

REFERENCES

1. M. A. Carreon, Porous crystals as membranes. *Science* **367**, 624–625 (2020).

2. P. Nugent, Y. Belmabkhout, S. D. Burd, A. J. Cairns, R. Luebke, K. Forrest, T. Pham, S. Q. Ma, B. Space, L. Wojtas, M. Eddaoudi, M. J. Zaworotko, Porous materials with optimal adsorption thermodynamics and kinetics for CO_2 separation. *Nature* **495**, 80–84 (2013).

3. D. S. Sholl, R. P. Lively. Seven chemical separations to change the world. *Nature* **532**, 435–437 (2016).

4. M. Elimelech, W. A. Phillip, The future of seawater desalination: Energy, technology, and the environment. *Science* **333**, 712–717 (2011).

5. M. A. Shannon, P. W. Bohn, M. Elimelech, J. G. Georgiadis, B. J. Mariñas, A. M. Mayes, Science and technology for water purification in the coming decades. *Nature* **452**, 301–310 (2008).

6. Z. Y. Li, H. T. Li, C. L. Jin, J. B. Li, J. J. Bao, X. P. Zhang, N. Zhang, G. H. He, C. Chen, Y. C. Song, Confined in situ synthesis of uniformly distributed mixed matrix membranes via solvent evaporation for efficient CO_2 capture. *Adv. Funct. Mater.* **35**, 2420713 (2024).

7. H. W. H. Lai, F. M. Benedetti, J. M. Ahn, A. M. Robeinson, Y. Wang, I. Pinnau, Z. P. Smith, Y. Xia, Hydrocarbon ladder polymers with ultrahigh permselectivity for membrane gas separations. *Science* **375**, 1390–1392 (2022).

8. I. Rose, C. G. Bezzu, M. Carta, B. Comesaña-Gándara, E. Lasseuguette, M. Chiara Ferrari, P. Bernardo, G. Clarizia, A. Fuoco, J. C. Jansen, K. E. Hart, T. P. Liyana-Arachchi, C. M. Colina, N. B. McKeownRose, Polymer ultrapermeability from the inefficient packing of 2D chains. *Nat. Mater.* **16**, 932–937 (2017).

9. L. F. Lei, F. J. Pan, A. Lindbråthen, X. P. Zhang, M. Hillestad, Y. Nie, L. Bai, X. Z. He, M. D. Guiver, Carbon hollow fiber membranes for a molecular sieve with precise-cutoff ultramicropores for superior hydrogen separation. *Nat. Commun.* **12**, 268 (2021).

10. R. Kukobat, M. Sakai, H. Tanaka, H. Otsuka, F. Vallejos-Burgos, C. Lastoskie, M. Matsukata, Y. Sasaki, K. Yoshida, T. Hayashi, K. Kaneko, Ultrapermeable 2D-channeled graphene-wrapped zeolite molecular sieving membranes for hydrogen separation. *Sci. Adv.* **8**, eabl3521 (2022).

11. S. C. Li, C. Ma, J. W. Hou, S. W. Yu, A. B. Chen, J. Du, P. A. Chater, D. S. Keeble, Z. H. Qiao, C. L. Zhong, D. A. Keen, Y. Liu, T. D. Bennett, Highly porous metal-organic framework glass design and application for gas separation membranes. *Nat. Commun.* **16**, 1622 (2025).

12. S. Q. Huang, S. X. Li, L. F. Villalobos, M. Dakhchoune, M. Micari, D. J. Babu, M. T. Vahdat, M. Mensi, E. Oveisi, K. V. Agrawal, Millisecond lattice gasification for high-density CO_2-and O_2-sieving nanopores in single-layer graphene. *Sci. Adv.* **7**, eabf0116 (2021).

13. K. J. Hsu, S. X. Li, M. Micari, H. Y. Chi, L. F. Villalobos, S. Q. Huang, L. P. Zhong, S. Q. Song, X. K. Duan, A. Züttel, K. V. Agrawal, Graphene membranes with pyridinic nitrogen at pore edges for high-performance CO_2 capture. *Nat. Energy* **9**, 964–974 (2024).

14. Z. Q. Yang, S. B. Poh, S. B. Xi, Y. Q. Liu, Q. X. Liu, D. Zhao, Packing engineering of zirconium metal-organic cages in mixed matrix membranes for CO_2/CH_4 separation. *Angew. Chem. Int. Ed. Engl.* **137**, e202418098 (2025).

15. L. M. Robeson, The upper bound revisited. *J. Membr. Sci.* **320**, 390–400 (2008).

16. H. B. Park, J. Kamcev, L. M. Robeson, M. Elimelech, B. D. Freeman, Maximizing the right stuff: The trade-off between membrane permeability and selectivity. *Science* **356**, eaab0530 (2017).

17. B. Comesaña-Gándara, J. Chen, C. G. Bezzu, M. Carta, I. Rose, M.-C. Ferrari, E. Esposito, A. Fuoco, J. C. Jansen, N. B. McKeown, Redefining the Robeson upper bounds for CO_2/CH_4 and CO_2/N_2 separations using a series of ultrapermeable benzotriptycene-based polymers of intrinsic microporosity. *Energy Environ. Sci.* **12**, 2733–2740 (2019).

18. K. Hazazi, Y. G. Wang, B. Ghanem, X. F. Hu, T. Puspasari, C. L. Chen, Y. Han, I. Pinnau, Precise molecular sieving of ethylene from ethane using triptycene-derived submicroporous carbon membranes. *Nat. Mater.* **22**, 1218–1226 (2023).

19. Y. W. Jia, Y. Q. Lu, H. Z. Yang, Y. Chen, F. Hillman, K. Y. Wang, C. Z. Liang, S. Zhang, Control of microporous structure in conjugated microporous polymer membranes for post-combustion carbon capture. *Adv. Funct. Mater.* **34**, 2407499 (2024).

20. Z. Y. Liu, W. L. Qiu, W. Y. Quan, W. J. Koros, Advanced carbon molecular sieve membranes derived from molecularly engineered cross-linkable copolyimide for gas separations. *Nat. Mater.* **22**, 109–116 (2023).

21. X. L. Chen, X. F. Fan, L. Wu, L. Z. Zhang, D. Guan, C. H. Ma, N. W. Li, Ultra-selective molecular-sieving gas separation membranes enabled by multi-covalent-crosslinking of microporous polymer blends. *Nat. Commun.* **12**, 6140 (2021).

22. Y. X. Ren, B. Y. Chong, W. Xu, Z. Q. Zhang, L. Liu, Y. Z. Wu, Y. T. Liu, H. F. Jiang, X. Liang, H. Wu, H. J. Zhang, B. J. Ye, C. L. Zhong, G. W. He, Z. Y. Jiang, Coordination-driven structure reconstruction in polymer of intrinsic microporosity membranes for efficient propylene/propane separation. *Innovation* **3**, 100334 (2022).

23. X. L. Chen, Z. G. Zhang, S. Xu, B. Zhang, Y. Qin, C. H. Ma, G. H. He, N. W. Li, Atomically distributed Al-F_3 nanoparticles towards precisely modulating pore size of carbon membranes for gas separation. *Nat. Commun.* **16**, 133 (2025).

24. L. Q. Hu, W. I. Lee, S. Roy, A. Subramanian, K. Kisslinger, L. X. Zhu, S. H. Fan, S. Hwang, V. T. Bui, T. Tran, G. Y. Zhang, Y. F. Ding, P. M. Ajayan, C. Y. Nam, H. Q. Lin, Hierarchically porous and single Zn atom-embedded carbon molecular sieves for H_2 separations. *Nat. Commun.* **15**, 5688 (2024).

25. X. Y. Tan, S. Robijns, R. Thür, Q. L. Ke, N. D. Witte, A. Lamaire, Y. Li, I. Aslam, D. V. Havere, T. Donckels, T. V. Assche, V. V. Speybroeck, M. Dusselier, I. Vankelecom, Truly combining the advantages of polymeric and zeolite membranes for gas separations. *Science* **378**, 1189–1194 (2022).

26. W. He, X. Z. Wang, J. Guan, Q. S. Liang, J. Ma, Y. Liu, W. W. Lim, C. W. Zhang, S. U. Hassan, H. J. Zhang, J. T. Liu, Membranes with molecular gatekeepers for efficient CO_2 capture and H_2 purification. *ACS Appl. Mater. Interfaces* **16**, 21222–21232 (2024).

27. W. K. Lai, Y. Jiao, Y. Liu, W. X. Fang, Z. G. Wang, M. D. Guiver, J. Jin, Engineering ultra-small Ag nanoparticles with enhanced activity in microporous polymer membranes for C_2H_4/C_2H_6 separation. *Adv. Mater.* **37**, e2416851 (2025).

28. J. Guan, J. C. Du, Q. Sun, W. He, J. Ma, S. U. Hassan, J. Wu, H. J. Zhang, S. Zhang, J. T. Liu, Metal-organic cages improving microporosity in polymeric membrane for superior CO_2 capture. *Sci. Adv.* **11**, eads0583 (2025).

29. X. L. Chen, G. N. Chen, C. Xie, L. Wu, G. P. Liu, N. W. Li, W. Q. Jin, Polyolefin reweaved ultra-micropore membrane for CO_2 capture. *Nat. Commun.* **16**, 282 (2025).

30. W. J. Koros, C. Zhang, Materials for next-generation molecularly selective synthetic membranes. *Nat. Mater.* **16**, 289–297 (2017).

31. B. Wang, Z. H. Qiao, J. Y. Xu, J. X. Wang, X. L. Liu, S. Zhao, Z. Wang, M. D. Guiver, Unobstructed ultrathin gas transport channels in composite membranes by interfacial self-assembly. *Adv. Mater.* **32**, e1907701 (2020).

32. L. Q. Hu, K. W. Chen, W. I. Lee, K. Kisslinger, C. Rumsey, S. H. Fan, V. T. Bui, N. Esmaeili, T. Tran, Y. F. Ding, M. Trebbin, C. Y. Nam, M. T. Swihart, H. Q. Lin, Palladium-percolated networks enabled by low loadings of branched nanorods for enhanced H_2 separations. *Adv. Mater.* **35**, e2301007 (2023).

33. A. Knebel, A. Bavykina, B. J. Datta, L. Sundermann, L. Garzon-Tovar, Y. Lebedev, S. Durini, R. Ahmad, S. M. Kozlov, G. Shterk, M. Karunakaran, I. D. Carja, D. Simic, I. Weilert, M. Klüppel, U. Giese, L. Cavallo, M. Rueping, M. Eddaoudi, J. Caro, J. Gascon, Solution processable metal-organic frameworks for mixed matrix membranes using porous liquids. *Nat. Mater.* **19**, 1346–1353 (2020).

34. Y. Wang, X. Ma, B. S. Ghanem, F. Alghunaimi, I. Pinnau, Y. Han, Polymers of intrinsic microporosity for energy-intensive membrane-based gas separations. *Mater. Today Nano* **3**, 69–95 (2018).

35. M. Chhetri, D. P. Leonard, S. Maurya, P. Sharan, Y. Kim, A. Kozhushner, L. Elbaz, N. Ghorbani, M. Rafiee, C. Kreller, Y. S. Kim, Electrochemical pumps based on ion-pair membranes for separation of hydrogen from low-concentration mixtures. *Nat. Energy* **9**, 1517–1528 (2024).

36. B. Zhu, S. S. He, Y. Yang, S. W. Li, C. H. Lau, S. M. Liu, L. Shao, Boosting membrane carbon capture via multifaceted polyphenol-mediated soldering. *Nat. Commun.* **14**, 1697 (2023).

37. A. T. Bridge, B. J. Pedretti, J. F. Brennecke, B. D. Freeman, Preparation of defect-free asymmetric gas separation membranes with dihydrolevoglucosenone (CyreneTM) as a greener polar aprotic solvent. *J. Membr. Sci.* **644**, 120173 (2022).

38. J. Ma, W. He, W. W. Lim, J. C. Du, Q. Sun, S. U. Hassan, D. Cao, J. Guan, J. T. Liu, Finely tailoring microstructure of hyperbranched polyimide membrane for facile natural gas upgrading. *J. Membr. Sci.* **720**, 123729 (2025).

39. B. Y. Gul, E. Pekgenc, V. Vatanpour, I. Koyuncu, A review of cellulose-based derivatives polymers in fabrication of gas separation membranes: Recent developments and challenges. *Carbohydr. Polym.* **321**, 121296 (2023).

40. Y. H. Cao, Z. Y. Liu, W. L. Qiu, W. J. Koros, Appealing sheath-core spun high-performance composite carbon molecular sieve membranes. *Angew. Chem. Int. Ed. Engl.* **62**, e202303915 (2023).

41. Y. C. Xiao, Y. Dai, T. S. Chung, M. D. Guiver, Effects of brominating Matrimid polyimide on the physical and gas transport properties of derived carbon membranes. *Macromolecules* **38**, 10042–10049 (2005).

42. S. Q. Cheng, Q. Lin, S. L. Li, Y. X. Guo, X. L. Han, Y. Sun, Y. Liu, Recent advancements in supramolecular macrocycles for two-dimensional membranes for separations. *Green Chem.* **25**, 7026–7040 (2023).

43. S. Q. Cheng, Y. Liu, Y. Sun, Macrocycle-based metal-organic and covalent organic framework membranes. *Coord. Chem. Rev.* **534**, 216559 (2025).

44. B. B. Zhang, J. Qiao, C. X. Dong, C. H. Yi, S. T. Qi, B. L. Yang, Dibenzo-21-crown-7-ether contained 6FDA-based polyimide membrane with improved gas selectivity. *Sep. Purif. Technol.* **264**, 118454 (2021).

45. J. T. Liu, Y. C. Xiao, T. S. Chung, Flexible thermally treated 3D PIM-CD molecular sieve membranes exceeding the upper bound line for propylene/propane separation. *J. Mater. Chem. A* **5**, 4583–4595 (2017).

46. W. He, X. Z. Wang, J. Guan, Q. S. Liang, J. Ma, Y. Liu, H. J. Zhang, C. W. Zhang, J. T. Liu, Mixed-matrix membranes with molecular recognition windows for selective helium extraction from natural gas. *Nat. Commun.* **17**, 2942 (2026).

47. J. Wu, C. Z. Liang, A. Naderi, T. S. Chung, Tunable supramolecular cavities molecularly homogenized in polymer membranes for ultraefficient precombustion CO_2 capture. *Adv. Mater.* **34**, 2105156 (2022).

48. S. J. Barrow, S. Kasera, M. J. Rowland, J. D. Barrio, O. A. Scherman, Cucurbituril-based molecular recognition. *Chem. rev.* **115**, 12320–12406 (2015).

49. D. A. Bulushev, G. N. Chekhova, V. I. Sobolev, A. L. Chuvilin, Y. V. Fedoseeva, O. A. Gerasko, A. V. Okotrub, L. G. Bulusheva, Cucurbit [6] uril as a co-catalyst for hydrogen production from formic acid. *Mater. Today Energy* **26**, 100998 (2022).

50. M. N. Lv, B. Li, Y. Y. Fu, Z. W. Wang, X. Z. Sun, J. X. Feng, Q. H. Zhang, D. J. Wan, J. Chen, Porous N-doped carbon with intercalation structure to disperse Fe/Fe_3C nanoparticles with CB6-based supramolecular assembly as carbon source towards ORR in zinc-air battery. *Int. J. Hydrogen Energy* **101**, 1077–1084 (2025).

51. Y. A. Patil, V. R. Mehta, D. R. Boraste, G. S. Shankarling, Facile preparation of Cucurbit[6] uril modified melamine sponge for efficient oil spill cleanup. *J. Environ. Chem. Eng.* **9**, 106603 (2021).

52. J. Wu, T. S. Chung, Supramolecular polymer network membranes with molecular-sieving nanocavities for efficient pre-combustion CO_2 capture, *Small Methods* **6**, 2101288 (2022).

53. H. Wu, L. O. Jones, Y. Wang, D. K. Shen, Z. C. Liu, L. Zhang, K. Cai, Y. Jiao, C. L. Stern, G. C. Schatz, J. F. Stoddart, High-efficiency gold recovery using cucurbit [6]uril. *ACS Appl. Mater. Interfaces* **12**, 38768–38777 (2020).

54. R. J. Wang, X. M. Wu, X. M. Yan, G. H. He, Z. W. Hu, Proton conductivity enhancement of SPEEK membrane through n-BuOH assisted self-organization. *J. Membr. Sci.* **479**, 46–54 (2015).

55. G. Z. Liu, B. Y. Mo, Y. A. Guo, Z. Y. Chu, X. M. Ren, K. C. Guan, R. J. Miao, Z. G. Wang, Y. X. Zhang, W. Q. Ji, G. P. Liu, H. Matsuyama, W. Q. Jin, Confined-coordination induced intergrowth of metal-organic frameworks into precise molecular sieving membranes. *Angew. Chem. Int. Ed. Engl.* **63**, e202405676 (2024).

56. M. Deng, J. Wei, J. D. Guo, Z. K. Qin, J. Song, J. F. Zheng, L. Yang, L. Yao, W. J. Liang, X. H. Ma, X. Z. He, J. D. He, J. J. Wang, Z. D. Dai, Cascade promotion of gas separation performances in CMS membranes: MOFs with functional groups and loaded noble metals. *Adv. Sci.* **12**, e03471 (2025).

57. Y. W. Xie, K. X. Lei, Y. H. Xiao, X. X. Huang, W. B. Li, De Novo mechanochemical MOF crystallization within polymers to design compatible mixed-matrix membranes for superior methane separation. *J. Membr. Sci.* **735**, 124583 (2025).

58. X. X. Huang, L. D. Chen, S. Z. Chen, P. C. Su, W. B. Li, Reconstruction of mixed matrix membranes by in situ vapor aminolysis for CO_2/N_2 and CH_4/N_2 separations. *J. Membr. Sci.* **685**, 121984 (2023).

59. G. Z. Liu, R. H. Li, X. Chen, L. Cheng, Y. Liu, G. P. Liu, W. Q. Jin, Pyrolysis temperature-regulated gas transport and aging properties in 6FDA-DAM polyimide-derived carbon molecular sieve membranes. *Sep. Purif. Technol.* **313**, 123459 (2023).

60. J. H. Qian, R. Y. Wang, H. G. Wu, F. C. Wang, M. Elimelech, Molecular simulations reveal gas transport mechanisms in polyamide membranes. *J. Membr. Sci.* **731**, 124056 (2025).

61. Y. Y. Zhang, M. Y. Sun, L. Li, R. S. Xu, Y. Q. Pan, T. H. Wang, Carbon molecular sieve/ZSM-5 mixed matrix membranes with enhanced gas separation performance and the performance recovery of the aging membranes. *J. Membr. Sci.* **660**, 120869 (2022).

62. J. H. Qian, R. Y. Wang, M. Elimelech, A universal metric for classifying gas transport regimes in nanoconfined media. *J. Chem. Phys.* **164**, 144904 (2026).

63. A. P. Thompson, H. M. Aktulga, R. Berger, D. S. Bolintineanu, W. M. Brown, P. S. Crozier, P. J. in 't Veld, A. Kohlmeyer, S. G. Moore, T. D. Nguyen, R. Shan, M. J. Stevens, J. Tranchida, C. Trott, S. J. Plimpton, LAMMPS—A flexible simulation tool for particle-based materials modeling at the atomic, meso, and continuum scales. *Comput. Phys. Commun.* **271**, 108171 (2022).

64. L. Martínez, R. Andrade, E. G. Birgin, J. M. Martínez, PACKMOL: A package for building initial configurations for molecular dynamics simulations. *J. Comput. Chem.* **30**, 2157–2164 (2009).

65. H. J. C. Berendsen, J. R. Grigera, T. P. Straatsma, The missing term in effective pair potentials. *J. Phys. Chem.* **91**, 6269–6271 (1987).

66. J. P. Ryckaert, G. Ciccotti, H. J. C. Berendsen, Numerical integration of a system with constraints: Of the cartesian equations of motion molecular dynamics of n-alkanes. *J. Comput. Phys.*, **23**, 327–341 (1977).

67. N. Nayir, A. C. T. V. Duin, S. Erkoc, Development of a ReaxFF reactive force field for interstitial oxygen in germanium and its application to GeO_2/Ge interfaces. *J. Phys. Chem. C* **123**, 1208–1218 (2018).

68. T. F. Willems, C. H. Rycroft, M. Kazi, J. C. Meza, M. Haranczyk, Algorithms and tools for high-throughput geometry-based analysis of crystalline porous materials. *Microporous Mesoporous Mater.* **149**, 134–141 (2012).

69. W. J. Koros, D. R. Paul, Design considerations for measurement of gas sorption in polymers by pressure decay. *J. Polym. Sci. Polym. Phys. Ed.* **14**, 1903–1907 (1976).

70. R. M. Barrer, Diffusivities in glassy polymers for the dual mode sorption model. *J. Membr. Sci.* **18**, 25–35 (1984).

71. E. V. Perez, K. J. Balkus Jr, J. P. Ferraris, I. H. Musselman, Mixed-matrix membranes containing MOF-5 for gas separations. *J. Membr. Sci.* **328**, 165–173 (2009).

72. F. Weigelt, P. Georgopanos, S. Shishatskiy, V. Filiz, T. Brinkmann, V. Abetz, Development and characterization of defect-free Matrimid® mixed-matrix membranes containing activated carbon particles for gas separation. *Polymer* **10**, 51 (2018).

73. Y. F. Zhang, K. J. Balkus Jr, I. H. Musselman, J. P. Ferraris, Mixed-matrix membranes composed of Matrimid® and mesoporous ZSM-5 nanoparticles. *J. Membr. Sci.* **325**, 28–39 (2008).

74. M. J. C. Ordoñez, K. J. Balkus Jr, J. P. Ferraris, I. H. Musselman, Molecular sieving realized with ZIF-8/Matrimid® mixed-matrix membranes. *J. Membr. Sci.* **361**, 28–37 (2010).

75. A. Mirzaei, A. H. Navarchian, S. Tangestaninejad. Mixed matrix membranes on the basis of Matrimid and palladium-zeolitic imidazolate framework for hydrogen separation. *Iran. Polym. J.* **29**, 479–491 (2020).

76. X. Jiang, S. S. He, G. Han, J. Long, S. W. Li, C. H. Lau, S. Zhang, L. Shao, Aqueous one-step modulation for synthesizing monodispersed ZIF-8 nanocrystals for mixed-matrix membrane. *ACS Appl. Mater. Interfaces* **13**, 11296–11305 (2021).

77. Y. F. Zhang, I. H. Musselman, J. P. Ferraris, K. J. Balkus, Jr, Gas permeability properties of mixed-matrix matrimid membranes containing a carbon aerogel: A material with both micropores and mesopores. *Ind. Eng. Chem. Res.* **47**, 2794–2802 (2008).

78. Z. V. Singh, L. L. Tan, M. G. Cowan, Y. W. Yang, W. Zhang, D. L. Gin, R. D. Noble, Pillar [5] arene/Matrimid™ materials for high-performance methane purification membranes. *J. Membr. Sci.* **539**, 224–228 (2017).

79. Y. B. Zhuang, J. G. Seong, Y. S. Do, W. H. Lee, M. J. Lee, M. D. Guiver, Y. M. Lee, High-strength, soluble polyimide membranes incorporating Tröger's Base for gas separation. *J. Membr. Sci.* **504**, 55–65 (2016).

80. J. J. Kong, J. J. Liu, P. Y. Jia, N. Qi, Z. Q. Chen, S. Xu, N. W. Li, Synergistic effect of thermal crosslinking and thermal rearrangement on free volume and gas separation properties of 6FDA based polyimide membranes studied by positron annihilation. *J. Membr. Sci.* **645**, 120163 (2022).

81. X. F. Hu, W. H. Lee, J. Y. Bae, J. S. Kim, J. T. Jung, H. H. Wang, H. J. Park, Y. M. Lee, Thermally rearranged polybenzoxazole copolymers incorporating Tröger's base for high flux gas separation membranes. *J. Membr. Sci.* **612**, 118437 (2020).

82. X. F. Hu, W. H. Lee, J. Y. Zhao, J. Y. Bae, J. S. Kim, Z. Wang, J. L. Yan, Y. B. Zhuang, Y. M. Lee, Tröger's Base (TB)-containing polyimide membranes derived from bio-based dianhydrides for gas separations. *J. Membr. Sci.* **610**, 118255 (2020).

83. B. Ghanem, N. Alaslai, X. H. Miao, I. Pinnau, Novel 6FDA-based polyimides derived from sterically hindered Tröger's base diamines: Synthesis and gas permeation properties. *Polymer* **96**, 13–19 (2016).

84. Y. B. Zhuang, J. G. Seong, Y. S. Do, W. H. Lee, M. J. Lee, Z. L. Cui, A. E. Lozano, M. D. Guiver, Y. M. Lee, Soluble, microporous, Tröger's Base copolyimides with tunable membrane performance for gas separation. *Chem. Commun.* **52**, 3817–3820 (2016).

85. R. Iwasa, T. Suizu, H. Yamaji, T. Yoshioka, K. Nagia, Gas separation in polyimide membranes with molecular sieve-like chemical/physical dual crosslink elements onto the top of surface. *J. Membr. Sci.* **550**, 80–90 (2018).

86. M. J. Li, Z. B. Zheng, Z. G. Zhang, N. W. Li, S. W. Liu, Z. G. Chi, J. R. Xu, Y. Zhang, "All polyimide" mixed matrix membranes for high performance gas separation. *Polymer* **13**, 1329 (2021).

87. S. N. Wijenayake, N. P. Panapitiya, S. H. Versteeg, C. N. Nguyen, S. Goel, K. J. Balkus, Jr, I. H. Musselman, J. P. Ferraris, Surface cross-linking of ZIF-8/polyimide mixed matrix membranes (MMMs) for gas separation. *Ind. Eng. Chem. Res.* **52**, 6991–7001 (2013).

88. S. W. Zhu, Z. G. Wang, Y. S. Shi, W. K. Lai, Y. T. Zhang, J. Y. Jin, J. Jin, Benzyl-induced crosslinking of polymer membranes for highly selective CO_2/CH_4 separation with enhanced stability. *Macromolecules* **55**, 6890–6900 (2022).

89. J. R. Wiegand, Z. P. Smith, Q. Liu, C. T. Patterson, B. D. Freeman, R. L. Guo, Synthesis and characterization of triptycene-based polyimides with tunable high fractional free volume for gas separation membranes. *J. Mater. Chem. A* **2**, 13309–13320 (2014).

90. S. H. Li, H. J. Jo, S. H. Han, C. H. Park, S. Kim, P. M. Budd, Y. M. Lee, Mechanically robust thermally rearranged (TR) polymer membranes with spirobisindane for gas separation. *J. Membr. Sci.* **434**, 137–147 (2013).

91. J. Ahn, W. J. Chung, I. Pinnau, M. D. Guiver, Polysulfone/silica nanoparticle mixed-matrix membranes for gas separation. *J. Membr. Sci.* **314**, 123–133 (2008).

92. Y. J. Wang, G. Yang, H. L. Guo, X. L. Meng, G. D. Kong, Z. X. Kang, R. Guillet-Nicolas, S. Mintova, Preparation of HKUST-1/PEI mixed-matrix membranes: Adsorption-diffusion coupling control of small gas molecules. *J. Membr. Sci.* **643**, 120070 (2022).

93. A. K. Itta, H. H. Tseng, M. Y. Wey, Fabrication and characterization of PPO/PVP blend carbon molecular sieve membranes for H_2/N_2 and H_2/CH_4 separation. *J. Membr. Sci.* **372**, 387–395 (2011).

94. X. Y. Meng, Y. C. Zhang, X. H. Zheng, C. Wang, S. J. Luo, N. W. Li, Y. F. Fan, Construction of thin selective layer on ZIF-8/PI MMMs via UV-induced reactions toward enhanced gas sieving properties. *J. Membr. Sci.* **730**, 124210 (2025).

95. F. Y. Li, Y. Li, T. S. Chung, S. Kawi, Facilitated transport by hybrid POSS®-Matrimid®-Zn^{2+} nanocomposite membranes for the separation of natural gas. *J. Membr. Sci.* **356**, 14–21 (2010).

96. D. Q. Vu, W. J. Koros, S. J. Miller. Mixed matrix membranes using carbon molecular sieves: I. Preparation and experimental results. *J. Membr. Sci.* **211**, 311–334 (2003).

97. M. Waqas Anjum, F. Vermoortele, A. L. Khan, B. Bueken, D. E. De Vos, I. F. J. Vankelecom, Modulated UiO-66-based mixed-matrix membranes for CO_2 separation. *ACS Appl. Mater. Interfaces* **7**, 25193–25201 (2015).

98. X. Y. Dong, Q. Liu, A. S. Huang. Highly permselective MIL-68 (Al)/matrimid mixed matrix membranes for CO_2/CH_4 separation. *J. Appl. Polym. Sci.* **133**, 43485 (2016).

99. M. Ahmadi, E. Taş, A. Kılıç, V. Kumbaracı, N. Talınlı, M. G. Ahunbay, Ş. B. Tantekin-Ersolmaz, Highly CO_2 selective microporous metal-imidazolate framework-based mixed matrix membranes. *ACS Appl. Mater. Interfaces* **9**, 35936–35946 (2017).

100. M. Peydayesh, S. Asarehpour, T. Mohammadi, O. Bakhtiari, Preparation and characterization of SAPO-34-Matrimid® 5218 mixed matrix membranes for CO_2/CH_4 separation. *Chem. Eng. Res. Des.* **91**, 1335–1342 (2013).

101. S. Shahid, K. Nijmeijer, Performance and plasticization behavior of polymer-MOF membranes for gas separation at elevated pressures. *J. Membr. Sci.* **470**, 166–177 (2014).

102. M. W. Anjum, F. De Clippel, J. Didden, A. L. Khan, S. Couck, G. V. Baron, J. F. M. Denayer, B. F. Sels, I. F. J. Vankelcome, Polyimide mixed matrix membranes for CO_2 separations using carbon-silica nanocomposite fillers. *J. Membr. Sci.* **495**, 121–129 (2015).

103. F. Moghadam, M. R. Omidkhah, E. Vasheghani-Farahani, M. Z. Pedram, F. Dorosti, The effect of TiO_2 nanoparticles on gas transport properties of Matrimid 5218-based mixed matrix membranes. *Sep. Purif. Technol.* **77**, 128–136 (2011).

104. J. Ahmad, M. B. Hägg, Development of matrimid/zeolite 4A mixed matrix membranes using low boiling point solvent. *Sep. Purif. Technol.* **115**, 190–197 (2013).

105. C. I. Chaidou, G. Pantoleontos, D. E. Koutsonikolas, S. P. Kaldis, G. P. Sakellaropoulos, Gas separation properties of polyimide-zeolite mixed matrix membranes. *Sep. Sci. Technol.* **47**, 950–962 (2012).

106. Q. L. Song, S. K. Nataraj, M. V. Roussenova, J. C. Tan, D. J. Hughes, W. Li, P. Bourgoin, M. A. Alam, A. K. Cheetham, S. A. Al-Muhtaseb, E. Sivaniah, Zeolitic imidazolate framework (ZIF-8) based polymer nanocomposite membranes for gas separation. *Energy Environ. Sci.* **5**, 8359–8369 (2012).

107. S. R. Venna, M. Lartey, T. Li, A. Spore, S. Kumar, H. B. Nulwala, D. R. Luebke, N. L. Rosi, E. Albenze, Fabrication of MMMs with improved gas separation properties using externally-functionalized MOF particles. *J. Mater. Chem. A* **3**, 5014–5022 (2015).

108. S. S. Hosseini, T. S. Chung, Carbon membranes from blends of PBI and polyimides for N_2/CH_4 and CO_2/CH_4 separation and hydrogen purification. *J. Membr. Sci.* **328**, 174–185 (2009).

109. J. Y. Liu, C. R. P. Fulong, L. Q. Hu, L. Huang, G. Y. Zhang, T. R. Cook, H. Q. Lin, Interpenetrating networks of mixed matrix materials comprising metal-organic polyhedra for membrane CO_2 capture. *J. Membr. Sci.* **606**, 118122 (2020).

110. D. Husken, T. Visser, M. Wessling, R. J. Gaymans, CO_2 permeation properties of poly (ethylene oxide)-based segmented block copolymers. *J. Membr. Sci.* **346**, 194–201 (2010).

111. N. C. Su, Z. P. Smith, B. D. Freeman, J. J. Urban, Size-dependent permeability deviations from Maxwell's model in hybrid cross-linked poly (ethylene glycol)/silica nanoparticle membranes. *Chem. Mater.* **27**, 2421–2429 (2015).

112. X. G. Li, I. Kresse, Z. K. Xu, J. Springer, Effect of temperature and pressure on gas transport in ethyl cellulose membrane. *Polymer* **42**, 6801–6810 (2001).

113. G. Perego, A. Roggero, R. Sisto, C. Valentini, Membranes for gas separation based on silylated polyphenylene oxide. *J. Membr. Sci.* **55**, 325–331 (1991).

114. W. J. Chen, C. R. Martin. Gas-transport properties of sulfonated polystyrenes. *J. Membr. Sci.* **95**, 51–61 (1994).

115. M. W. Hellums, W. J. Koros, G. R. Husk, D. R. Paul, Fluorinated polycarbonates for gas separation applications. *J. Membr. Sci.* **46**, 93–112 (1989).

116. J. S. McHattie, W. J. Koros, D. R. Paul, Gas transport properties of polysulphones: 1. Role of symmetry of methyl group placement on bisphenol rings. *Polymer* **32**, 840–850 (1991).

117. J. T. Li, K. Nagai, T. Nakagawa, S. C. Wang, Preparation of polyethyleneglycol (PEG) and cellulose acetate (CA) blend membranes and their gas permeabilities. *J. Appl. Polym. Sci.* **58**, 1455–1463 (1995).

118. L. Hao, P. Li, T. S. Chung, PIM-1 as an organic filler to enhance the gas separation performance of Ultem polyetherimide. *J. Membr. Sci.* **453**, 614–623 (2014).

119. S. Y. Hwang, W. S. Chi, S. J. Lee, S. H. Im, J. H. Kim, J. Kim, Hollow ZIF-8 nanoparticles improve the permeability of mixed matrix membranes for CO_2/CH_4 gas separation. *J. Membr. Sci.* **480**, 11–19 (2015).

120. C. J. Duan, X. M. Jie, D. D. Liu, Y. M. Cao, Q. Yuan, Post-treatment effect on gas separation property of mixed matrix membranes containing metal organic frameworks. *J. Membr. Sci.* **466**, 92–102 (2014).
